# Integrated Proteomics and Metabolomics of Safflower Petal Wilting and Seed Development

**DOI:** 10.3390/biom14040414

**Published:** 2024-03-28

**Authors:** Delphine Vincent, Priyanka Reddy, Daniel Isenegger

**Affiliations:** Agriculture Victoria Research, AgriBio, Centre for AgriBioscience, 5 Ring Road, Bundoora, VIC 3083, Australia; priyanka.reddy@agriculture.vic.gov.au (P.R.); daniel.isenegger@agriculture.vic.gov.au (D.I.)

**Keywords:** multi-omics, integrated analysis, lipophilic, bottom-up proteome, non-polar metabolome

## Abstract

Safflower (*Carthamus tinctorius* L.) is an ancient oilseed crop of interest due to its diversity of end-use industrial and food products. Proteomic and metabolomic profiling of its organs during seed development, which can provide further insights on seed quality attributes to assist in variety and product development, has not yet been undertaken. In this study, an integrated proteome and metabolic analysis have shown a high complexity of lipophilic proteins and metabolites differentially expressed across organs and tissues during seed development and petal wilting. We demonstrated that these approaches successfully discriminated safflower reproductive organs and developmental stages with the identification of 2179 unique compounds and 3043 peptides matching 724 unique proteins. A comparison between cotyledon and husk tissues revealed the complementarity of using both technologies, with husks mostly featuring metabolites (99%), while cotyledons predominantly yielded peptides (90%). This provided a more complete picture of mechanisms discriminating the seed envelope from what it protected. Furthermore, we showed distinct molecular signatures of petal wilting and colour transition, seed growth, and maturation. We revealed the molecular makeup shift occurring during petal colour transition and wilting, as well as the importance of benzenoids, phenylpropanoids, flavonoids, and pigments. Finally, our study emphasizes that the biochemical mechanisms implicated in the growing and maturing of safflower seeds are complex and far-reaching, as evidenced by AraCyc, PaintOmics, and MetaboAnalyst mapping capabilities. This study provides a new resource for functional knowledge of safflower seed and potentially further enables the precision development of novel products and safflower varieties with biotechnology and molecular farming applications.

## 1. Introduction

Safflower (*Carthamus tinctorius* L.) is an erect annual herbaceous plant of the Asteraceae family that originated from the eastern Mediterranean coast and was domesticated more than 4500 years ago [1]. Native to arid regions of the Middle East, it has a remarkable adaptability to a wide range of climatic conditions, from semi-arid to temperate zones. It is an ideal crop for arid to semi-arid agricultural land with limited water availability and relatively high temperatures; its deep tap root system with abundant thin horizontal roots allows the plant to extract water and nutrients from deeper layers of soil than many other crop plants [2]. Safflower’s ability to withstand salinity, drought, strong winds, hailstorms, and flooding has made its cultivation possible in diversified environments. Furthermore, its relatively short growth cycle, typically ranging from 100 to 150 days, allows flexibility in crop rotation strategies, thus enhancing soil fertility and reducing disease pressure. Accordingly, *C. tinctorius* is currently widely grown in more than 60 countries and regions on all continents but Antarctica [3]. The thistle-like plants range in height from 0.3 to 1.5 m and harbour extensive branching ending with globular flowering heads of vibrant colour shades from white, to yellow, orange, or red [2]. Each plant produces 3–50 capitula, each containing 20–180 florets, ultimately yielding 15–60 achenes [4]. A thick hull protects the cotyledon inside the seed, which starts to mature 4–5 weeks after flowering.

Safflower is a multipurpose crop. It is mainly cultivated for its petals and oil content in seeds used as edible cooking oil, food colouring, fabric dyes, cosmetics, animal and birdfeed, medicines, pharmaceuticals, biofuel, and lubricant [3,5]. Asian traditional herbal medicine employs flowers and seeds to treat gynaecological, cardiovascular, and cerebrovascular diseases as well as blood stasis and osteoporosis [6]. Also known as fake saffron, *C. tinctorius* flowers have commonly been used as a cheaper substitute for saffron, and methods have been devised to authenticate spices and detect known adulterants [7,8,9]. Safflower capitula are a rich source of bioactive substances, with 200 substances identified thus far [10]. With a moisture content of 4.7%, the composition of safflower petals is 1.82% protein, 4.8% lipids, 11.6% crude fibre, and 10.8% ash. Abundant petal compounds comprise alkaloids, flavonoids, lignanoids, organic acids and polyacetylenes, alkanediols, riboflavin, steroids, and quinochalcone C-glycosides [10]. Flavonoids of the C-glucosylquinochalcone group constitute the bulk of petal pigments, including carthamine (safflower yellow, carthamus red) and carthamidin (carthamic acid). Safflower pigments have become so important that syntheses of their analogues have been optimised [11], and more recently, flavonoid extraction has been refined [12]. Most of the pharmacological activities of *C. tinctorius* can be attributed to flavonoids and alkaloids, especially the quinochalcone c-glycoside hydroxysafflor yellow A (HSYA), N-(p-Coumaroyl)serotonin, and N-feruloylserotonin [6]. HSYA was reported to exhibit significant biological activity in the treatment of coronary heart disease, myocardial infarction, ischaemic encephalopathy, cerebral thrombosis, and stroke [1].

*C. tinctorius* is an oilseed crop whose oil content is in the range of 23–40%, which is on par with that of sunflower, olive, and peanut [13]. The applications of safflower oil seeds in industrial, pharmaceutical, and food products depend upon their fatty acid (FA) composition, which fluctuates among plant species, cultivars, and growing conditions [2]. Safflower oil contains a high proportion of unsaturated FAs for medicinal as well as dietetic purposes. The most abundant ones are linoleic and oleic acid comprising 77.9–79.5% and 9.5–11.3% of total Fas, respectively. Saturated fatty acids are present in lower proportions (9.7–10.8% of total FAs); the prominent ones are palmitic and stearic acids representing 7.2–8.6% and 2.0–2.4%, respectively. A resurgence in the demand for renewable plant-based oils has rekindled interest in safflower due to its high-quality oil yields and genotypic variation in FA composition, with a focus on linoleic, oleic, and stearic acids [5]. Interestingly, super high oleic (~90%) genetically modified varieties have been developed and commercialised and are highly suitable as industrial lubricants among other renewable products [14].

The safflower genome is diploid and contains 24 chromosomes [15]. It was recently sequenced with 33,343 gene models predicted [16] and 82,916 gene products annotated [17]. *C. tinctorius* is the closest relative in the wild, and its single progenitor was *C. palaestinus*, which was domesticated in the Levant region [15]. Its closest relative with a sequenced genome is the globe artichoke (*Cynara cardunculus*) [18]. Safflower harbours uniquely expanded gene families involved in lipid metabolism and transport, as well as abscisic acid signaling [16]. The 47 genes responsible for lipid biosynthesis were identified from a collection of 605 safflower germplasms [19]. Notably, the fatty acid desaturase 2 (FAD2) and chalcone synthase (CHS) families, which function in the FA and flavonoid biosynthesis pathways, respectively, were expanded via tandem duplications in safflower. The FAD2 family with 11 genes is exceptionally large in safflower, including the seed-specific FAD2 oleate Δ12 desaturase genes responsible for converting stearic acid to oleic acid [20].

The availability of annotated genes has spurred post-genomics research on safflower. Temporal transcriptome profiling of developing seeds revealed that FAs were actively synthesised from 10 to 14 days after flowering (DAF) and degraded after 18 DAF [21]. The main genes implicated were stearoyl-[acyl-carrier-protein] 9-desaturase gene (SAD) from 10 to 14 DAF and oleate desaturase (FAD2–1) from 14 to 18 DAF, with the latter being regulated by 13 candidate transcription factors. Proteomics studies have aimed at comparing the response to drought stress and re-watering between cultivated and wild young plants [22], observing the impact of growth regulators on salt stress responses in seedlings [23], and establishing the composition, functional, and antioxidant properties of flour [24]. Metabolomics was applied to monitor the changes in pigment composition during the blooming period [25] and to assess how betaine salvage seedling growth suppression under salt stress [26]. The integration of several post-genomics workflows as a multi-omics strategy has successfully advanced the understanding of safflower capitula biology, in particular, white floret formation [27], as well as flavonoid profiling during colour transition [28], colour variation [29], or methyl jasmonate treatment [30]. An integrated proteome and lipidome analysis of naturally aged safflower seeds varying in vitality indicated that enzymes involved in glycerolipid metabolism and FA degradation contributed to the degradation of oil bodies and membrane lipids and are thus responsible for a decline in seed vigour during natural seed ageing [31].

Whilst post-genomics research has garnered interest in safflower, there is little to no knowledge and investigation to elucidate the molecular mechanisms involved in achene development. The present work provides a new fundamental resource that incorporates a comprehensive proteomics and metabolomics approach to temporally profile seeds collected at five key development stages covering filling and maturation. We have also dissected fully mature seeds into husks and cotyledons to show unique molecular signatures. Furthermore, in the early seed development stages, we sampled flowers as they wilted and transitioned colours to better understand which lipophilic proteins and compounds facilitated these biochemical processes.

## 2. Materials and Methods

### 2.1. Safflower Cultivation, Sampling, and Storage

Safflower plants (cv. S317) were grown from seed in 200 mm pots filled with commercial potting media (Biogro, VIC, Australia). in greenhouse conditions maintained at 20–24 °C and a 14 h photoperiod supplemented by high-pressure sodium lamps. Plants were maintained in a high health condition. 

**Figure 1:**
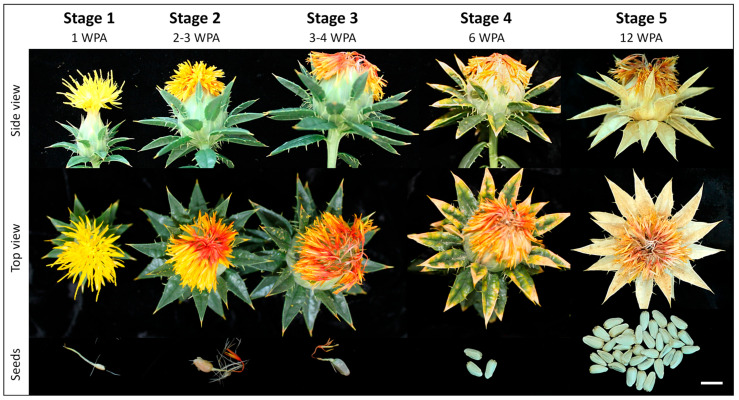
Developmental stages of collected safflower inflorescences and seeds. WPA: weeks post-anthesis, scale bar 10 mm.

The sampling of seeds was done by harvesting whole capitula at various developmental stages estimated from the number of weeks from floret and anther emergence and floret morphology, such as colour (yellow to red) and the level of senescence/wilting (Figure 1). 

A minimum of 3 capitula were removed from plants at each developmental stage and were carefully dissected using a scalpel blade to obtain seed/achene samples. Five developmental stages were targeted and reported as weeks post anthesis (WPA): stage 1 corresponded to 1 WPA, stage 2 corresponded to 2–3 WPA, stage 3 corresponded to 3–4 WPA, stage 4 corresponded to 6 WPA, and the ultimate stage 5 at 12 WPA marker full maturity of the oil seeds. 

**Figure 2:**
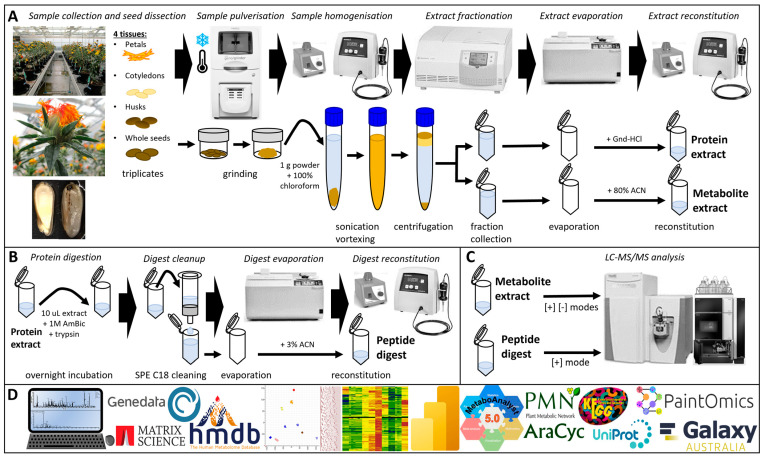
Sample preparation and analysis. (**A**) Sample extraction; (**B**) protein digestion for BUP workflow; (**C**) LC-MS/MS analysis of peptide digest and metabolite extract; (**D**) data analysis/mining.

Up to 10 undamaged seeds/achenes with florets at stages 1–3 (1–4 WPA) were sampled and placed into a 2 mL tube. At later stages 4 and 5 (6 and 12 WPA), individual seeds were larger, and 5 were transferred to 2 mL collection tubes. All samples were collected in triplicate, snap-frozen in liquid nitrogen, and stored at −80 °C. The subsequent processing steps are summarised in Figure 2.

### 2.2. Sample Preparation

Frozen collected samples were transferred into a −80 °C prechilled 50 mL grinding jar with two 8 mm and two 3 mm −80 °C prechilled metal grinding balls. The grinding jars were immediately placed into −80 °C prechilled metal racks and adapted into an automated tissue homogeniser and cell lyser (Geno/Grinder^®^ 2010, SPEX SamplePrep, Metuchen, NJ, USA). The samples were pulverised for 2 min at 1750 rpm. 

An amount of 1 g (whole seeds and cotyledons) or 200 mg (husks and petals) of frozen ground material was transferred into a 15 mL tube and 9 mL of 100% chloroform was added. Tubes were incubated in a sonicator bath for 15 min, vortexed for 1 min, and further resuspended using an MS 1.5 sonicator probe (Ultrasonic Homogeniser SONOPULS mini 20, Bandelin, Berlin, Germany) for 30 s with 90% amplitude. This step was repeated following the addition of 4 mL 100% chloroform. 

Tubes were centrifuged using a swing bucket rotor for 10 min at 5000 rpm (Sigma centrifuge 4–16 KS, Osterode am Harz, Germany). Floating material was scooped out and discarded. The chloroform phase was equally divided into two tubes for each proteomics and metabolomics stream and completely evaporated using a vacuum centrifuge (SPD-2010 SpeedVac, ThermoFisher Scientific, Scoresby, VIC, Australia) without heat. 

### 2.3. Bottom-up Proteomics

#### 2.3.1. Protein Extraction and Digestion

To the 2 mL tubes containing evaporated chloroform phase, 0.5 mL Gnd-HCl buffer (6 M Guanidine hydrochloride, 0.1 M Bis-Tris, 10 mM DTT, 5.37 mM sodium citrate tribasic dihydrate) was added and probe-sonicated for 30 s with 90% amplitude. The strong chaotropic and reducing conditions allowed for efficient denaturation of lipophilic proteins, thereby amenable to solubilisation in our water-based buffer. The tubes were thoroughly vortexed and incubated for 60 min at 60 °C. The tubes were left to cool to room temperature for 5 min, and 10 µL of 1 M iodoacetamide was added. The tubes were vortexed for 30 s and incubated in the dark for 30 min. The tubes were then centrifuged at 13,000 rpm for 15 min.

Protein trypsin/Lys-C digestion, peptide clean-up and digest reconstitution steps were performed as described [32,33]. Briefly, 10 µL of protein extract was transferred into a tube, diluted six times in 50 mM ammonium bicarbonate, and 1 µg enzyme was added for overnight incubation at 37 °C. Digests were desalted using solid phase extraction, eluted in 250 µL 80% ACN/0.1% FA/water, fully evaporated using a vacuum centrifuge, and reconstituted in 100 µL of 0.1% FA/water which matched our LC starting conditions. 

#### 2.3.2. Liquid Chromatography (LC) and Mass Spectrometry (MS) of Peptides

The equipment and consumables used for LC-MS and LC-MS/MS analyses were detailed [32,33]. The LC flow rate was 0.2 mL/min with an autosampler and oven temperatures of, respectively, 10 °C and 60 °C. Mobile phase A consisted of 0.1% FA in water, and mobile phase B contained 0.1% FA in ACN. A 5 µL of tryptic digest was injected and LC-separated for 60 min along the following gradient: 3% B for 2.5 min, 3–40% B gradient for 37.5 min, increased up to 98% B gradient for 3 min, 98% B for 8 min, drop down to 3% B in 1 min, and 3% B for 8 min.

For LC-MS analyses, spectra were acquired using the full MS scan mode of the Fourier transform (FT) orbitrap mass analyser (FTMS) in positive ion mode at a resolution of 15,000 along a 300–2000 *m*/*z* mass window in profile mode with 3 micro-scans.

For LC-MS/MS analyses based on the Nth order double play method in data dependant mode, two scan events were created, one full FTMS scan as specified above followed by a full ion trap scan (ITMS) in positive ion mode along a 300–2000 *m*/*z* mass window in centroid mode with 4 micro-scans. Ignoring singly charged ions, the 10 most abundant peaks and a minimum signal threshold of 5000 were fragmented using collision-induced dissociation (CID) with a normalised collision energy of 35%, 0.25 activation Q, and activation time of 10 ms. The precursor isolation width was 2 *m*/*z*. Dynamic exclusion was activated, and peptides selected for fragmentation more than once within 10 s were excluded from selection for 20 s.

#### 2.3.3. Protein Database and Mascot Identification

Protein sequences from various sources were downloaded: safflower genome sequencing project [16] (71,896 entries, http://safflower.scuec.edu.cn/, downloaded on 5 December 2023), Uniprot *C. tinctorius* proteins (304 entries, https://www.uniprot.org/taxonomy/4222, accessed on 12 November 2022), and Uniprot *Cardueae* tribe (35,708 entries, https://www.uniprot.org/taxonomy/219103, accessed on 12 November 2022); a contaminant database was also retrieved (common Repository of Adventitious Proteins (cRAP); https://ftp.thegpm.org/fasta/cRAP, downloaded in March 2022). A single fasta protein database containing all aforementioned protein sequences with duplicated amino acid (AA) sequences removed and their reversed decoys was created using the Galaxy workflow consigned in [33]. This fasta file included 143,792 sequences; it was imported and parsed into our Mascot server.

LC-MS/MS RAW files were exported as MGF using the MSconvertGUI free tool (https://proteowizard.sourceforge.io/tools/msconvert.html, [34]). All MGF files were combined into a single file by using the Galaxy tool to concatenate datasets tail-to-head (cat). 

Using Mascot (version 2.6.2, Matrix Science Ltd., London, UK), the MGF file was searched against the fasta DB described above with the following parameters: MS/MS ions search, Mascot generic data format, ESI-TRAP instrument, monoisotopic masses, trypsin enzyme, up to 9 missed cleavages, carbamidomethyl (C) as fixed modification, oxidation (M) as variable modifications, quantitation none, monoisotopic mass, 2+, 3+ and 4+ peptide charge, 20 ppm peptide tolerance, 0.5 Da MS/MS tolerance, and error-tolerant search, which allows the matching of uninterpreted MS/MS data and identifying unexpected modifications [35] (for more information on error tolerant searching, the reader is advised to read https://www.matrixscience.com/help/error_tolerant_help.html). The search result with a significance threshold of *p* < 0.1 was exported as a CSV file.

### 2.4. Metabolomics

#### 2.4.1. Metabolite Extraction

Evaporated chloroform samples were reconstituted in 1 mL 80% ACN/water with thorough vortexing for 5 min. Tubes were centrifuged and 0.1 mL supernatant was transferred into vials for LC-MS analysis.

#### 2.4.2. LC-MS Analysis of Metabolites

For untargeted metabolite profiling, a Vanquish ultra-high performance liquid chromatography (UHPLC) system (Thermo Fisher Scientific, Bremen, Germany) with a binary pump, autosampler, and temperature-controlled column compartment, coupled with a QExactive (QE) Plus mass spectrometer (Thermo Fisher Scientific, Bremen, Germany) with electrospray (ESI) probe operating in both positive and negative modes, was used. Prior to data acquisition, the system was calibrated with Pierce LTQ Velos ESI positive and negative ion calibration solution (Thermo Fisher Scientific). Spectrometry data were acquired using Thermo Xcalibur V. 2.1 (Thermo Fisher Scientific Inc., Waltham, MA, USA). Nitrogen was used as the sheath, auxiliary, and sweep gases at flow rates of 28, 15, and 4 L/min, respectively. Spray voltage was set at 4000 V (positive and negative). A Thermo Fisher Scientific Hypersil Gold 1.9 μm, 100 mm × 2.1 mm column with a gradient mobile phase consisting of 0.1% formic acid in H_2_O (A) and 0.1% formic acid in acetonitrile (B), at a flow rate of 0.3 mL/min was used. The gradient began at 2% B, increasing to 100% B over 11 min, followed by 4 min at 100% B before a 5 min equilibration with 2% B.

MS cycles were composed of 1 full MS scan and up to 10 full-scan MS/data-dependent MS2 (ddMS2) events. The top 10 cycles triggered an MS2 event at the peak apex with an isolation window of 0.4 *m*/*z*. A 5.0 s delay was required for the same ion to trigger a new MS2 event (dynamic exclusion). For MS data acquisition, positive and negative ion data were captured over a mass range of 80–1200 *m*/*z*, with a mass resolution set at 35,000 (full width at half maximum, FWHM, at *m*/*z* 200). The automatic gain control (AGC) target was 3 × 10^6^ and the maximum injection time (IT) was 200 ms. For MS/MS data acquisition, ddMS2 in both positive and negative ionisation modes were set over a mass range of 80–1200 *m*/*z*, with a mass resolution of 17,500. The AGC target was 1 × 10^5^ and the maximum IT was 50 ms. Ions were fragmented with stepped collision energy (20, 40 and 60%).

#### 2.4.3. Metabolite Identification

Safflower metabolite identification was carried out in Genedata Refiner using LC-MS data searched against the Human Metabolome Database (HMDB Version 5.0, [36]), which contains 220,945 metabolite entries including both water-soluble and lipid-soluble metabolites. 

### 2.5. Quantitation and Statistical Analyses

LC-MS and LC-MS/MS RAW files from peptide and metabolites experiments were processed in the Genedata Expressionist Refiner module (version 16, Genedata AG, Basel, Switzerland) as explained in [33,37]. The datasets obtained presented features in rows and samples in columns; missing values were blanks.

The quantitative data generated by the Refiner module for both proteomics and metabolomics was combined in Excel by adding unique identifiers. The combined data was imported into the Genedata Expressionist Analyst module to perform the statistical analyses. The quantities were normalised using sample weights and autoscaled per feature. The distribution of feature quantities across samples was displayed using box plots.

Several unsupervised multivariate clustering methods were employed. A principal component analysis (PCA) was performed using 50% valid values and a covariance matrix. A k-means analysis was completed on the full dataset using 50% valid values, 16 clusters, 50 maximum iterations, and positive correlation distances. Using the k-means clusters that displayed petal or seed specificity, two self-organising maps analyses were carried out across developmental stages of whole seeds using 3 clusters as well as on wilting petals using 2 clusters. The same parameters were applied throughout: 50% valid values, 50 maximum iterations, and positive correlations.

Three univariate analyses were completed. A linear model (LM) was performed on petals using time series as a covariate factor and applied to the k-means clusters that displayed petal specificity. Another LM was performed on seeds also with time series as a covariate factor and applied to the k-means clusters which displayed seed specificity. A comparison of cotyledons and husks was achieved using a *t*-test on k-means clusters specific to each tissue using 50% valid values, 10 repeat bootstraps, and balanced permutations. *p*-values were charted against fold change as a volcano plot.

### 2.6. Data Visualisation, Data Mining, and Bioinformatics

The metabolites’ MHDB identifiers were uploaded into the ID conversion tool of MetaboAnalyst (https://www.metaboanalyst.ca/MetaboAnalyst/upload/ConvertView.xhtml) [38] to retrieve metabolites descriptions including KEGG compound identifiers (COs), as well as InChlKeys. The latter were uploaded into the ClassyFire online tool (https://cfb.fiehnlab.ucdavis.edu/; [39]) to categorise known metabolites. Outputs were exported to Power BI and plotted as histograms, pies, and stacked column charts.

A total of 136,350 protein FASTA sequences from *Arabidopsis thaliana* were downloaded from Uniprot using Taxonomy 3202 (https://www.uniprot.org/taxonomy/3702) [40]. The file was uploaded to Galaxy Australia (Galaxy version 2.14.1, https://usegalaxy.org.au/) and converted into a database using the “NCBI BLAST+ makeblastdb” tool [41]. The FASTA sequences of safflower proteins identified in this study were searched in Galaxy Australia against the *A. thaliana* database using the “NCBI BLAST+ blastp” tool [41] with the following parameters: blastp type, evalue of 0.0001, BLOSUM45 scoring matrix, default gap costs, 1 maximum hit, and 30% minimum query coverage. 

UniprotKB ID mapping (https://www.uniprot.org/id-mapping) [40] was used to upload and retrieve the full description of all identified protein accessions including *A. thaliana* blastp hits. Thus, geneIDs used in MetaboAnalysts, PaintOmics, and AraCyc, FASTA sequence used in KEGG, as well as gene ontology (GO) terms and IDs, were recovered. GO IDs and counts were uploaded in Revigo (http://revigo.irb.hr/; [42]) with the following parameters: large list, higher value is better, *A. thaliana* as a species, and SimRel semantic similarity measure. Outputs were exported to Power BI and plotted as scatterplots, treemaps, stacked column charts, and treemap bar charts.

The *A. thaliana* protein FASTA sequences were uploaded online in the KEGG BlastKOALA tool (https://www.kegg.jp/kegg/mapper/assign_ko.html) [43] against the Brassicaceae family (taxonomy 3700) to retrieve KEGG orthologs (KOs). Metabolite COs and protein KOs were uploaded into the KEGG search tool (https://www.kegg.jp/kegg/mapper/search.html) by specifying the *A. thaliana* organism code (ath) [44].

The joint-pathway analysis module of MetaboAnalyst 5.0 was used online (https://www.metaboanalyst.ca/) to map both *A. thaliana* geneIDs and metabolite HMDB codes [38]. The parameters were as follows: integrated metabolic pathways as the pathway database, enrichment analysis using Fisher’s exact test, degree centrality for topology measure, and combined *p*-values at the pathway level as an integration method.

Proteomics and metabolomics quantitative datasets with *A. thaliana* geneIDs and metabolite names were up-loaded into PaintOmics (version 4, https://www.paintomics.org/) [45] by choosing *A. thaliana* as an organism and selecting KEGG, Reactome, and MapMan as databases. Raw quantities were used, and missing values were replaced with 0.01 values.

The same quantitative datasets employed in PaintOmics were combined into one file to be analysed into AraCyc from Plant Metabolics Network [46] (https://pmn.plantcyc.org/organism-summary?object=ARA). The file was uploaded into the cellular overview/OMICS viewer by specifying the use of any of the known identifiers and absolute values and selecting both the cellular overview diagram and the omics dashboard. The cellular overview dynamic animation was recorded using the Chrome extension Veed.io.

All Excel spreadsheets describing samples identified peptides and metabolites, quantitative data, and statistical results were uploaded to the PowerBI desktop for data merging, filtering, and visualisation (treemaps, scatterplots, violin plots, donut charts, histograms, and word clouds).

## 3. Results and Discussion

### 3.1. Proteomics and Metabolomics Successfully Discriminated Safflower Organs and Developmental Stages

**Figure 3:**
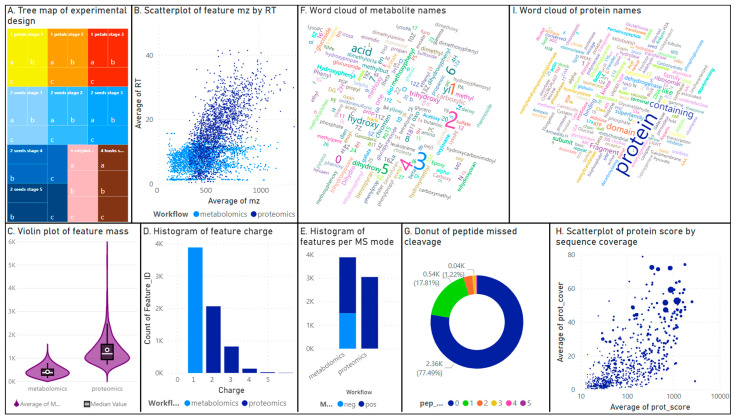
Charting proteomics and metabolomics outputs in the Power BI desktop dashboard.

Our multifactorial experimental design explored both tissue development and specificity by sampling petals and seeds over time (Figure 1) and comparing cotyledons and husks. The seed developmental timeline was split into five stages, from very immature at 1 WPA to full maturity at 12 WPA. The petals were sampled in stages 1–3, thus marked by a colour change from yellow to orange-red and wilting. As petals were fully dry past 5 WPA, we did not collect them on stages 4–5 to maintain protein integrity. In all, 10 tissues were collected in triplicates (Supplementary Table S1 and Figure 3A); the resulting 30 samples were processed to recover lipophilic proteins and metabolites (Figure 2). Tryptic peptides and organic compounds were separated by LC-MS along 20 and 40 min gradients, spanning *m*/*z* 300–1500 and 80–1200, respectively (Figure 3B). Metabolites were singly charged with masses ranging from 82 to 1197; peptides hosted 2–5 positive charges with masses distributed from 599 to 5585 (Figure 3C,D). 

All metabolomics identification results were captured in Supplementary Table S2. The number of metabolites identified using negative and positive MS modes were 1500 and 2385, respectively (Figure 3E), and corresponded to 2179 unique compounds, including 396 that were identified in both modes. Charting the compound names as a word cloud highlighted prominent group labels, such as acid (358 occurrences) (Figure 3F), denoting the prevalence of fatty acids in safflower oily seeds. Other frequent terms included hydroxy, di-, tri-hydroxy (228, 119, and 103 occurrences, respectively), or carboxylic (102 instances). ClassyFire classification highlighted that most compounds (47%) belonged to the lipids and lipid-like molecules superclass, with 481 fatty acyls, 458 prenol lipids, 299 glycerophospholipids, 249 steroids, and 80 glycerolipids (Supplementary Figure S1A). The second most abundant superclass comprised 18% phenylpropanoids and polyketides; they included 281 flavonoids, 99 coumarins, 80 cinnamic acids, and 80 linear 1,3-diarylpropanoids. The third largest superclass contained benzenoids (9%), with 231 benzenes and derivatives, 80 phenols, and 51 naphtalenes. 

All proteomics identification results including decoy hits are captured in Supplementary Tables S3A,B. A total of 3043 peptides were identified (Figure 3E) and matched 724 unique proteins. Up to five missed cleavages were found by the Mascot algorithm (Figure 3G). Most identified peptides (2358, 77%) did not feature any missed cleavage; 542 (18%) peptides contained two missed cleavages, and 94 (3%) had three missed cleavages. At the protein level, the Mascot score ranged from 13 to 2937 with up to 79% of the AA sequence covered (Figure 3H). A word cloud of protein names illustrated the high frequency of common terms featured in identity descriptions such as “protein” (151 occurrences), “containing” (49 instances), “domain” (41 occurrences), “fragment” (26 instances), “family” (22 items), or “binding” (14) (Figure 3I). Putting those aside revealed 17 ribosomal proteins, 9 histones, 13 dehydrogenases, 13 kinases, and 7 oxidases, along with many other enzymes. Revigo classification of proteins revealed that most peptides originated from proteins involved in seed maturation (42%) and exhibited a nutrient reservoir activity (28%) (Supplementary Figure S1B). Other prominent biological processes (BP) were proteolysis (9%), glycolytic processes (7%), and translation (5%). These large GOBP categories bore no semantic similarities as can be seen on the scatterplot (Supplementary Figure S1B). Cellular component proportions were more balanced: nucleus 11%, cytoplasm 7%, extracellular region 7%, and membrane 7%. A total of 2002 (66%) peptides presented post-translational modifications (PTMs, Supplementary Table S3A). The most frequent modifications were Gln->Lys (Q) (478/2002, 24%), followed by oxidation (265/2002, 13%) and methylation (131/2002, 7%). An error-tolerant parameter was allowed during the Mascot algorithm search to identify PTMs other than carbamidomethylation and oxidation. However, caution must be exercised when interpreting the identification results so that only proteins that already have at least one significant peptide match should incorporate new matches [35]. Indeed, even though our sample preparation method did not include a labelling step, label modifications were also attributed to 6% of our identified peptides because of the error-tolerant search. Those label modifications should be disregarded, yet we left them to make the community aware of the limitations of such a method. This warrants follow-up experiments to validate the PTMs identified in this work such as the two-pronged strategy suggested by [47], namely, the verification of identified modifications in the initial dataset and targeted experiments using synthetic peptides.

**Figure 4:**
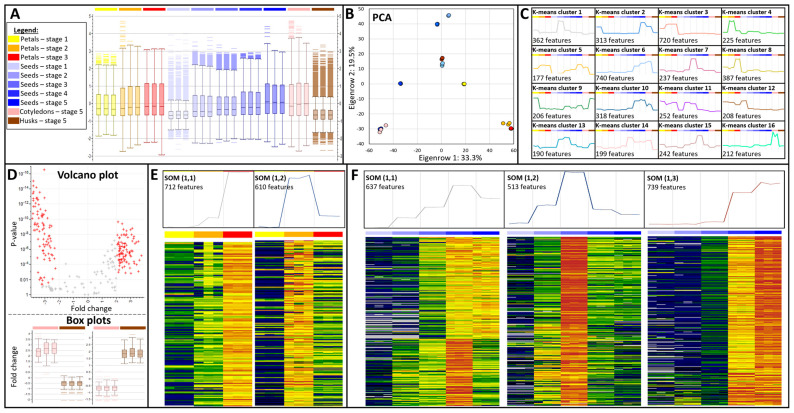
Statistical analyses. (**A**) Box plot of quantitative data per sample. The legend in panel A applies to all panels; (**B**) PCA plot of all samples; (**C**) 16 K-mean clusters across all samples; (**D**) volcano plot displaying *t*-test output of cotyledons vs. husks; significant features with both a fold change > 2 and a *p*-value < 0.05 are highlighted in red. Below are box plot charts of significant features either up-regulated in cotyledons (left chart) or down-regulated in cotyledons (right chart); (**E**) SOM and heat map of petal wilting; (**F**) SOM and heat map of developing seeds. The colours in the heat maps in panels (**E**,**F**) correspond to low abundance in dark blue and high abundance in dark red.

Quantitative data generated from both metabolomics and proteomics streams were combined into a single dataset containing 6917 features across 30 samples, which was normalised prior to statistical analyses. A box plot chart highlighted the dynamic range variation across tissues, with stage 1 seeds and hulls displaying the shortest interquartile ranges, while petals at stages 2–3 and seed and cotyledons at stage 5 showed the largest interquartile range (Figure 4A). The high reproducibility of the workflow was demonstrated by the very similar box plots across triplicates. It was confirmed by PCA that triplicates either overlaid one another or grouped together (Figure 4B). PC1 explained 33.3% of the variance (2303 features) and separated petals on the right-hand side from seeds/cotyledons on the left. Husk and stage 1 seeds are located in the middle of PC1, thus they do not contribute. PC2 explained 19.5% of the variance (1348 features) and aligned with developmental stages. Stages 2–5 of whole seeds covered the whole PC2 axis from top to bottom, whilst stages 1 to 3 of petals were sequentially distributed along the bottom half. This PCA biplot illustrates that quantifying peptides and metabolites from chloroform fraction faithfully captured the experimental design by discriminating tissues over time. 

The whole dataset was subjected to k-means clustering and grouped into 16 clusters, explaining 94% of the variance overall. Cluster size ranged from 190 (cluster 13) to 740 (cluster 6). Many clusters gathered features that accumulated in a single sample type such as clusters 1, 7, or 15, respectively, displaying a pick of expression in seeds at stages 2, 3, and 4 (Figure 4C). Likewise, k-mean cluster 16 depicted 212 features unique to cotyledons. Other clusters were less specific with up-regulation across several samples; this was exemplified in cluster 5 highlighting the gradual upregulation of 177 features over developmental stages in petals, seeds, and cotyledons. There was no cluster unique to hull samples; cluster 9 grouped 206 features accumulating in both husks and yellow petals. K-means clusters 3, 4, and 8 were unique to petals and were combined into 1332 features for further analyses to characterise petal colour change and wilting. Likewise, features specific to seeds/cotyledons and found in k-means clusters 6, 7, 10, 13, 14, and 15 were combined (1926 features) for subsequent analyses of seed maturation. 

### 3.2. Comparison of Cotyledon and Husk Reveals the Complementarity of Metabolomics and Proteomics

A safflower plant typically yields 1000–2500 seeds, with mature seeds reaching 6–10 mm in length and protected by a thick hull representing about 45% of the total seed content in recent varieties [48]. One of our aims in this study was to compare cotyledons and husks by identifying their molecular signatures. We performed a *t*-test on the 418 features listed in k-means clusters 9 and 16 (Figure 4C), which were the most specific to those tissues; then we plotted the fold changes against *p*-values as a volcano chart (Figure 4D). Choosing an arbitrary *p*-value significance of 0.05 and fold change of 2 listed 92 and 94 features up-regulated in cotyledons and hulls, respectively (Supplementary Tables S2 and S3). Bar plots of those significant peptides and metabolites confirmed the opposite expression patterns displayed by each set. 

Husk-induced analytes were predominantly metabolites (92/94), with only two peptides listed. One of the peptides (BUP_Peak_053987) belonged to unannotated *C. tinctorius* proteins (CtAH06T0128300.2_AMAGQIR) that blasted an *A. thaliana* mitochondrial ATP synthase subunit O involved in oxidative phosphorylation (K02137). The other peptide (BUP_Peak_083816) was also from an unannotated safflower protein (CtAH07T0252400.1_EINSLAK) whose AA sequence aligned against *A. thaliana* dihydrolipoyllysine-residue succinyltransferase (EC 2.3.1.61). This enzyme acts in the TCA cycle and the lipoic acid metabolism (K00658). Many of the 92 metabolites accumulating in hulls were lipids and lipid-like molecules (35%), phenylpropanoids and polyketides (21%), or organoheterocyclic compounds (20%). The most significantly up-regulated compounds were lisuride (MET-neg_Group_0839), which is an indoloquinoline alkaloid also present in Ginko biloba (Itil et al. 1998), followed by octadecanedioic acid (MET-neg_Group_0709), also named stearic acid, the most common fatty acid. Two glycerophospholipids PA(15:0/22:2(13Z,16Z)) (MET-neg_Group_2614) and PA(20:1(11Z)/15:0) (MET-neg_Group_2503) also accumulated in hull. Several serotonin derivatives were over-expressed in husks, including N-(p-coumaroyl) serotonin, which was also isolated from safflower seed meal [49].

Cotyledon-induced analytes were predominantly peptides (84/92), with only eight metabolites listed. Three of those were lipids including oleamide (MET-pos_Group_1940), which was three times up-regulated in cotyledons; this FA amide was identified from purslane seed extract [50]. One flavonoid, 3,4,trihydroxy-{[hydroxy-(hydroxy-methoxyphenyl)-oxo-4H-chromen-yl]oxy}oxane-carboxylic acid (MET-neg_Group_1523), accumulated 2.74 times more in cotyledons than in hulls. This was confirmed with a similar expression profile by peptide BUP_Peak_270832 from chalcone-flavonone isomerase, an important enzyme for flavonoid biosynthesis that catalyzes the intramolecular cyclization of chalcones into (S)-flavanones. Other accumulating compounds were an AA derivative (N-lactoyGlycine, MET-neg_Group_0003) and a dipeptide (prolyproline, MET-pos_Group_0164). One-third of husk-induced peptides (26/84) originated from 12S seed storage proteins CRA1 involved in seed maturation (GO:0010431)/nutrient reservoir activity (GO:0045735). In *A. thaliana*, 12S seed storage proteins accumulated once cell elongation processes had finalised in developing seeds [51]. Other cotyledon-abundant peptides came from ribosomal proteins acting in translation, peptidases involved in proteolysis, or kinase driving phosphorylation, as well as structural proteins located in the cytoskeleton or cell walls.

Our multi-omics results show that hulls mostly featured metabolites (99%), while cotyledons mostly featured peptides (90%). This demonstrated the complementarity of metabolomics and proteomics providing a more complete picture of mechanisms discriminating the seed envelope from what it protected.

### 3.3. Petal Molecular Signature Shifts during Colour Transition and Wilting

The second objective of our study was to identify the molecular signatures of petal wilting and colour transition. We combined the 1322 petal-specific features (747 metabolites and 575 peptides) listed in k-means clusters 3, 4, and 8 (Figure 4C). The largest proportion (360/747, 48%) of metabolites induced in petals belonged to the lipids and lipid-like molecule superclass, including 161 fatty acyls, 114 prenol lipids, 38 steroids, and 34 glycerophospholipids (Supplementary Figure S2D). This was substantiated by 20 peptides matching proteins involved in lipid metabolism and transport. Two non-specific lipid-transfer proteins were identified in this work (nsLTP1 and nsLTP10). NsLTPs are small extracellular proteins that only exist in land plants, bind hydrophobic molecules, and are associated with multiple processes [52]. Elevated nsLTP gene expression was reported in the petal and sepal abscission zone, where lipophilic substances are deposited to form the protective layer [53]. Another LTP, chorein -N motif protein (CtAH11T0254500.1), employs its extended hydrophobic channel to simultaneously bind dozens of lipids and facilitate their passage through the cell membrane to the cytosol [54]. 

The second most prominent superclass of petal compounds was benzenoids (106/747, 14%), which constitute the most widespread plant fragrances and substantially contribute to total floral scent [55]. Their roles include pollinator attraction, plant–plant communication, and herbivore repellent [56]. Among the 62 benzenes and derivatives, we identified benzoic acid (MET-pos_Group_0968), hydroxybenzoic acid (MET-neg_Group_0236), benzaldehyde (MET-pos_Group_0891), and many benzene derivatives that are the hallmark of scent constituents. Those benzenoid compounds originating from the trans-cinnamic acid branch of the general phenylpropanoid pathway and lacking the three-carbon chain are volatile and thereby not particularly amenable to LC-MS analysis. This warrants further studies using suitable analytical technology such as GC-MS to validate those metabolites. Moreover, we did not identify enzymes participating in benzenoid metabolism. 

The third most frequent superclass of petal compounds was phenylpropanoids and polyketides (87/747, 12%), including 29 flavonoids like heterophyllin (MET-neg_Group_1641) and cycloartocarpin (MET-neg_Group_1351), 17 cinnamic acids such as sinapine (MET-neg_Group_0685), as well as 9 coumarins. Synthesized from phenylpropanoid derivatives, flavonoids are a major class of plant secondary metabolites that serve a multitude of functions including tissue pigmentation and antioxidant activity. Over 60 flavonoids have been isolated from safflower [1]. 

We performed a self-organising map (SOM) to identify two expression patterns across the 1322 petal-specific features and generated heat maps of the profiles underlining each trend. Moreover, we carried out a linear model (LM) to establish the significance of the feature expression pattern over time (Supplementary Tables S2 and S3). The first SOM cluster (1,1) displayed an increase in abundance of 712 features (457 metabolites and 255 peptides) from stage 1 (1 WPA) to stage 3 (3–4 WPA) (Figure 4E), when the petals turned red and wilted but were not completely dry (Figure 1). The SOM group (1,1) listed numerous flavonoids specifically accumulating throughout wilting, including chromen-one derivatives (MET-neg_Group_0813, MET-neg_Group_2139, and MET-neg_Group_2424) (MET-neg_Group_2530) and 3,3’-Dihydroxy-4’,5,trimethoxyflavan (MET-neg_Group_2531). Although no enzymes involved in flavonoid metabolism were found in this SOM cluster, we detected peptides from phenylpropanoid-related gene products. The unannotated safflower protein CtAH06T0045000.1 (BUP_Peak_298915 and BUP_Peak_029589) matched *A. thaliana* Dirigent protein 21 (DIR21), which during lignan biosynthesis mediates regio- and stereoselectivity of bimolecular phenoxy radical coupling [57]. The acyl carrier protein-like protein (A0A118JU17_CYNCS, BUP_Peak_220692) exhibited a 4-coumarate--CoA ligase activity (4CL EC 6.2.1.12), and its abundance gradually increased during the petal colour transition from yellow and orange to red. Furthermore, 4CL is a key phenylpropanoid pathway enzyme by biosynthesising monolignol through the production of flavonoid precursor p-coumaroyl-CoA. In addition, 4CL safflower transcripts were down-regulated in white flowers relative to red flowers [27].

The second SOM cluster (1,2) marked a peak of expression in wilting orange petals (2–3 WPA) for 610 features (290 metabolites and 320 peptides). In our study, analytes were much less abundant in yellow petals than during the later stages. Treemap bar charts showed that proteins accumulating in petals were mostly involved in glycolytic processes (GO:0006096), phosphorylation (GO:0016310), and pectin catabolitic processes (GO:0045490) (Supplementary Figure S2A). They were predominantly extracellular (GO:0005576), cytoskeletal (GO:0005856), or nuclear (GO:0005634) proteins whose activities involved ATP binding (GO:0005524) and ATP synthase (GO:0046933 and GO:0046961).

Focusing on significant features (*p*-values < 0.05) with the shortest distances in each SOM group highlighted the change in molecular signatures occurring during the petal wilting process. Indeed, proteins accumulating in orange petals acted in proteolysis (GO:0006508), located in the mitochondrion (GO:0005739), and bore a cysteine-type peptidase activity (GO:0008234) (Supplementary Figure S2B). The most significant peptide of SOM cluster (1,2) (BUP_Peak_082043) matched *A. thaliana* mitochondrial aldehyde dehydrogenase family 2 member B4 (ALDH2a, EC 1.2.1.3, Q9SU63), which is an ATP binding enzyme. Conversely, proteins accumulating in red petals acted in glycolysis (GO:0006096), located in the cytoplasm (GO:0005737), and featured an ATP-binding activity (GO:0005524) (Supplementary Figure S2C). The most significant peptide of SOM cluster (1,1) (BUP_Peak_157663) matched *A. thaliana* dihydrolipoyllysine-residue succinyltransferase (EC 2.3.1.61, A0A178V2M2_ARATH), which participates in many pathways. The metabolite signature also shifted with more flavonoids, coumarins, carboxylic acids, and benzopyrans in wilting orange petals (Supplementary Figure S2E) and more fatty acyls, prenol lipids, steroids, benzenes, and phenols in wilted red petals (Supplementary Figure S2F). All safflower-specific pigments identified in this study were found in the SOM cluster (1,2) and accumulated in wilting orange petals (Supplementary Figure S2G). They were safflor yellow B (MET-neg_Group_3373), anhydrosafflor yellow B (MET-pos_Group_4993), carthamin (MET-neg_Group_3153), and safflomin C (MET-neg_Group_2188 and MET-pos_Group_0699). Similar abundance profiles were reported by Pu and colleagues [25] in their study on safflower blooming. Pigments missing in our dataset were HSYA, safflor yellow A, and isosafflomin C; their water-solubility might have prohibited their extraction under our organic conditions [1]. Safflower pigments were reported to be more abundant in red inflorescences than white inflorescences [27]. Safflower flowers are known adulterants of saffron and detection methods have been devised [8,9]. Our list of identified metabolites and proteins specific to petals could be further used as safflower biomarkers to test commercial saffron samples. 

### 3.4. Safflower Seed Growth and Maturation Are Driven by a Complex Tapestry of Biochemical Mechanisms

The final aim of our study was to study safflower development, which could be summarily divided into seed filling/growth (stages 1–3) and seed maturation (stages 4–5). Achene size rapidly expanded during the first collection time points, from 5 mm at 1 WPA, to 9 mm at 2–3 WPA and reaching their final length of 11 mm after 3–4 WPA (Figure 1). Hulls acquired their last shiny grey colouring at 6 WPA. We combined the 1889 seed-specific features listed in k-means clusters 6, 7, 10, 13, 14, and 15 (Figure 4C); they comprised 1239 peptides and 650 metabolites. With 364 (29%) entries, seed maturation (GO:0010431) represented the largest Gene Ontology Biological Process (GOBP) category of the seed proteome, followed by translation (GO:0006412, 63 occurrences), proteolysis (GO:0005608, 61 entries), and glycolytic processes (GO:0003096, 35 entries) (Supplementary Figure S3A). The most frequent Gene Ontology Cellular Component (GOCC) terms were nucleus (GO:0005634, 54 occurrences), ribonucleoprotein complex (GO:19990904, 53 entries), ribosome (GO:0005840, 51 entries), and membrane (GO:0016020, 44 occurrences). The largest Gene Ontology Molecular Function (GOMF) class in seeds was nutrient reservoir activity (GO:0045735) with 388 (31%) occurrences, followed by ATP binding activity (GO:0005524, 92 entries), and structural constituent of ribosome (GO:0003735, 37 entries). Multiple occurrences of 12S seed storage protein CRD, 12S seed storage protein CRA1, and late embryogenesis abundant (LEA) proteins (LEA31, BUP_Peak_073034 Supplementary Figure S3I) constituted the bulk of safflower seed proteome. Lipids and lipid-like molecules predominated the seed metabolome with 237 (36%) (Supplementary Figure S3E) and consisted of 11% fatty acyls, 8% prenol lipids, 7% glycerophospholipids, and 3% glycerolipids. Confirming the large lipid component of oily safflower seeds, numerous proteins involved in FA and lipid pathways were identified in this study, such as a lipoxygenase 2 (CtAH11T0245300.1), a bifunctional inhibitor/plant lipid transfer protein/seed storage helical domain-containing protein (A0A103YGV1_CYNCS), a putative vacuolar protein sorting-associated protein (DUF1162) (CtAH11T0254500.1), a lipid transfer protein 4 (CtAH10T0004400.1), and an oleosin 3 (A0A7R6LUT3_CARTI) (Supplementary Table S3A). The second largest superclass was represented by 160 (25%) phenylpropanoids and polyketides, including 7% flavonoids, 2% isoflavonoids, 1% 2-arylbenzofuran flavonoids, 3% linear 1,3-diarylpropanoids, 3% coumarins, 2% cinnamic acids, and 1% diarylheptanoids. This was substantiated at the protein level with the accumulation during seed maturation of 4CL and DIR21, as well as a beta-xylosidase 1 (CtAH11T0219400.1, BUP_Peak_191007 and BUP_Peak_119513, Supplementary Figure S3I), which belonged to the seed coat development category (GO:0010214). The third superclass listed 80 (12%) benzenoids, with 6% benzenes, 3% phenols, 1% phenol esthers, and 1% napthalenes. Other superclasses consisted of 62 (10%) organoheterocyclic compounds, 39 (6%) organic acids, 28 (4%) organic oxygen compounds, and 19 (3%) lignans. 

We performed a SOM across the 1,889 seed-specific features to produce three patterns, along with heat maps of the profiles underlining each trend. An LM isolated features whose abundance significantly changed over time (Supplementary Tables S2 and S3). The first SOM cluster (1,1) grouped 637 features (253 metabolites and 384 peptides) displaying a gradual increase in abundance up to stage 4, followed by a slight dip at full maturity, stage 5 (Figure 4F). The second SOM cluster (1,2) was the smallest and comprised 513 features (255 metabolites and 258 peptides) presenting a bell-shaped expression profile, peaking at stage 3. The third SOM cluster (1,3) was the largest with 739 features (142 metabolites and 597 peptides) and showed almost no accumulation from stages 1 to 3, followed by a sharp increase in abundance during stages 4–5, when the seeds reached their full size and maturity (Figure 1). Focusing on significant features (*p*-values < 0.05) with the shortest distances in each SOM group highlighted the change in the proteome and metabolome of maturing seeds. Most proteins yielding peptides listed in SOM clusters (1,1) and (1,3) and significantly accumulating in fully grown seeds (stages 4–5) belonged to the seed maturation category (GO:0010431) and displayed nutrient reservoir activity (GO:0045735) (Supplementary Figure S3B,D). In the same SOM clusters, phenylpropanoids and lipids dominated (Supplementary Figure S3F,H). Peptides from oleosin 3 (BUP_Peak_195471, BUP_Peak_195474, and BUP_Peak_100441) accumulated during the maturation phase (Supplementary Figure S3I). Oleosins are structural proteins found in vascular plant organs characterised with high oil content that undergo extreme desiccation as part of their maturation process, such as seeds; they help stabilize oil bodies [58]. The abundance of lipoxygenase 2 (LOX2; BUP_Peak_012862, BUP_Peak_121717, BUP_Peak_177481, BUP_Peak_181300) increased considerably during stage 4 and achieved a very high level of expression during stage 5 (Supplementary Figure S3I). Plant LOXs display an oxygenase activity towards either linoleic acid or linolenic acid and may be involved in a number of diverse aspects of plant physiology, including growth and development. During the first step in the biosynthesis of oxylipins, LOXs catalyze the oxygenation of polyunsaturated fatty acids [59]. In our study, alpha-linolenic acid (MET-pos_Group_1916), and linoleic acid (MET-pos_Group_1934) steadily accumulated throughout seed filling stages, reaching their apex and very high abundance particularly for alpha-linolenic acid at stage 4 and becoming less abundant at full maturity (Supplementary Figure S3I). Linoleic acid (MET-pos_Group_1934) and oleic acid (MET-neg_Group_0573, MET-pos_Group_0296, MET-pos_Group_0297, and MET-pos_Group_0298) exhibited the same expression pattern, while the content of their precursors, palmitic acid (MET-neg_Group_0498) and stearic acid (MET-neg_Group_0585), accumulated during seed filling (Supplementary Table S2). Translation (GO:0006412) and structural constituent of ribosome (GO:0003735) classified the proteins significantly peaking during stage 3 in the SOM cluster (1,2) (Supplementary Figure S3C). Only a few compounds significantly marked stage 3 in seeds, particularly phenylpropanoids (Supplementary Figure S3G), such as the prenylated flavone albanin H (MET-neg_Group_3002) (Supplementary Table S2). Our results suggested that safflower seed growth was sustained by active translational and lignification mechanisms until the full size was achieved, following which seed storage and oil body production processes predominated.

**Figure 5:**
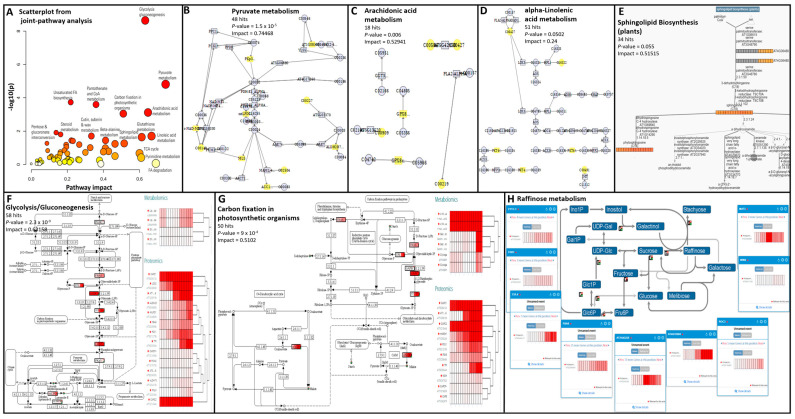
Data mining reveals key mechanisms in safflower seed development. (**A**–**D**) MetaboAnalyst joint-pathway analysis; (**E**) AraCyc cellular overview and omics dashboard; (**F**–**H**) PaintOmics.

To integrate quantitative results of identified peptides and metabolites, we employed three free online multiomics-compatible resources, namely, MetaboAnalyst, AraCyc, and PaintOmics. Whilst AraCyc [60] had been used to map identified biomarkers from multi-omics plant experiments [61,62], to our knowledge, MetaboAnalyst joint-pathway analysis and PaintOmics [63] have never been applied to plant datasets. The joint pathway analysis module of MetaboAnalyst [64] simultaneously analyzed gene products and metabolites (KEGG or HMDB) of interest within the context of metabolic pathways. We used HMDB identifiers and *A. thaliana* official gene symbols for safflower metabolites and proteins, respectively, along with *p*-values. Overall, 66 pathways were flagged, incorporating 6 to 141 identifiers mapped in the network explorer view (Supplementary Figure S4). The best-covered pathways were purine metabolism, FA biosynthesis, phenylpropanoid biosynthesis, amino sugar, and nucleotide sugar metabolism, as well as flavonoid biosynthesis (Supplementary Table S4). AraCyc Omics viewer (PMN https://pmn.plantcyc.org/organism-summary?object=ARA 15 November 2023) accepted metabolite names and *A. thaliana* gene names, along with quantitative data for each 15 seed samples in a single file. A total of 306 analytes were recognised (113 compounds and 193 proteins); 148 of those could not be assigned to a pathway (Supplementary Table S5). A total of 34 pathways were highlighted; the best represented were the secondary metabolite biosynthesis, followed by nucleoside and nucleotide biosynthesis and AA biosynthesis/degradation. The expression profiles of the 158 mapped analytes could be viewed dynamically in the cellular overview at the highest level (Supplementary Video S1 and Supplementary Figure S5A) or at a much finer level by zooming in, as exemplified on the pyrimidine salvage pathway (Supplementary Figure S5B). The AraCyc Omics dashboard was complementary to the cellular overview as it displayed expression profiles averaged per pathway at either the highest level possible (Supplementary Figure S5C) or, as illustrated on the TCA cycle, zoomed in at the next level down (Supplementary Figure S5D) all the way to the biochemical reaction level (Supplementary Figure S5E). The web tool PaintOmics integrates multiple omic datasets onto KEGG, Reactome, and MapMan biological pathway maps. Our metabolomics and proteomics datasets were uploaded independently using compound names and *A. thaliana* gene names along with quantitative data for each 15 seed samples. Recognising up to 169 identifiers, 122 (104 KEGG and 18 MapMan) pathways were flagged representing 78% cellular processes and 11% genetic information processes (Supplementary Figure S6A). The best-covered pathways were the biosynthesis of secondary metabolites, AA metabolism, and raffinose metabolism (Supplementary Table S6). PaintOmics depicted a complex enrichment map (Supplementary Figure S6B). Unlike the MetaboAnalyst joint pathway analysis module, both AraCyc and PaintOmics could interpret quantitative data and incorporate expression profiles in visualisations. Both MetaboAnalyst and PaintOmics interpretations of safflower seed proteome and metabolome were faithful to the GO and ClassyFire categorisations detailed above. The MetaboAnalyst scatterplot of pathway impact against *p*-values afforded a summarised view of the biological processes enriched in safflower seeds (Figure 5A). The most impactful pathways were glycolysis/glucogenesis (Figure 5F) followed by pyruvate metabolism (Figure 5B) and carbon fixation in plants (Figure 5G), both of which were reviewed to be essential to seed processes [65] and quality [66]. Raffinose metabolism was also well covered with the identification of numerous enzymes involved in the production of soluble sugars (Figure 5H). No water-soluble sugars were identified in this study as an organic solvent was employed during extraction. Raffinose was reported to be the most concentrated soluble sugar in safflower seeds, thereby contributing to seed desiccation and storability [13]. Other impactful pathways were arachidonic acid, alpha-linoleic acid, and sphingolipid metabolisms (Figure 5C–E). For instance, dehydrophytosphingosine (MET-pos_Group_0356) and sphinganine (MET-pos_Group_2072) showed a gradual increase as safflower seeds filled and matured.

## 4. Conclusions

In this study, we have explored the lipophilic proteome and metabolome of various safflower reproductive tissues to determine the distinct molecular signatures of petal wilting and colour transition, seed growth, and maturation, as well as a comparison between the developing cotyledon and the husk. We demonstrated that proteomics and metabolomics successfully discriminated safflower reproductive organs and developmental stages with the identification of 2179 unique compounds and 3043 peptides matching 724 unique proteins. The comparison between the developing cotyledon and husk revealed the complementarity of both technologies, as some tissues yielded mostly proteins (cotyledons) whilst others yielded compounds (hull). We revealed the molecular makeup shift occurring during petal colour transition and wilting, as well as the importance of benzenoids, phenylpropanoids, flavonoids, and pigments. Finally, our study emphasizes that the biochemical mechanisms implicated in the growing and maturing of safflower seeds are complex and far-reaching, as evidenced by AraCyc, PaintOmics, and MetaboAnalyst mapping capabilities. Future studies might include studying the hydrophilic protein and metabolite fractions of safflower seeds, cotyledons, hulls, and petals and comparing them to the results reported in the present work. 

## Data Availability

All the data is available in the Supplementary Files.

## Supplementary Materials

The following supporting information can be downloaded at https://www.mdpi.com/article/10.3390/biom14040414/s1, Supplementary Figure S1: Classification of metabolites using ClassyFire and of peptides using gene ontology and REVIGO. Supplementary Figure S2: Data mining reveals molecular shifts in safflower wilting petals. Supplementary Figure S3: Data mining reveals molecular shifts in developing seeds up to full maturity. Supplementary Figure S4: MetaboAnalyst joint-pathway analysis; network explorer view highlighting the pathways enriched during safflower seed development. Supplementary Figure S5: AraCyc omics data analysis mapping the pathways involved in safflower seed development. Supplementary Figure S6: PaintOmics analysis. KEGG pathway classification displays the pathways involved in safflower seed development. Supplementary Video S1: Dynamic expression profiling of the 158 analytes mapped in AraCyc cellular overview. Supplementary Table S1: Safflower sample description. Supplementary Table S2: Safflower metabolite description, statistical, and quantitation results. Supplementary Table S3A: Safflower peptide description, statistical, and quantitation results. Supplementary Table S3B: Decoy hits from proteomics. Supplementary Table S4: MetaboAnalyst joint-pathway analysis results in safflower seeds. Supplementary Table S5: AraCyc Omics viewer results in safflower seeds. Supplementary Table S6: PaintOmics results in safflower seeds.

## References

[1] Xian B., Wang R., Jiang H., Zhou Y., Yan J., Huang X., Chen J., Wu Q., Chen C., Xi Z. (2022). Comprehensive Review of Two Groups of Flavonoids in *Carthamus tinctorius* L. Biomed. Pharmacother..

[2] Khalid N., Khan R.S., Hussain M.I., Farooq M., Ahmad A., Ahmed I. (2017). A Comprehensive Characterisation of Safflower Oil for Its Potential Applications as a Bioactive Food Ingredient—A Review. Trends Food Sci. Technol..

[3] Mani V., Lee S.-K., Yeo Y., Hahn B.-S. (2020). A Metabolic Perspective and Opportunities in Pharmacologically Important Safflower. Metabolites.

[4] Singh R.J. (2007). Genetic Resources, Chromosome Engineering, and Crop Improvement: Oilseed Crops.

[5] Thoday-Kennedy E., Banerjee B., Panozzo J., Maharjan P., Hudson D., Spangenberg G., Hayden M., Kant S. (2023). Dissecting Physiological and Agronomic Diversity in Safflower Populations Using Proximal Phenotyping. Agriculture.

[6] Zhang L.-L., Tian K., Tang Z.-H., Chen X.-J., Bian Z.-X., Wang Y.-T., Lu J.-J. (2016). Phytochemistry and Pharmacology of *Carthamus tinctorius* L. Am. J. Chin. Med..

[7] Hegazi N.M., Khattab A.R., Frolov A., Wessjohann L.A., Farag M.A. (2022). Authentication of Saffron Spice Accessions from Its Common Substitutes via a Multiplex Approach of UV/VIS Fingerprints and UPLC/MS Using Molecular Networking and Chemometrics. Food Chem..

[8] Ryparova Kvirencova J., Navratilova K., Hrbek V., Hajslova J. (2023). Detection of Botanical Adulterants in Saffron Powder. Anal. Bioanal. Chem..

[9] Paredi G., Raboni S., Marchesani F., Ordoudi S.A., Tsimidou M.Z., Mozzarelli A. (2016). Insight of Saffron Proteome by Gel-Electrophoresis. Molecules.

[10] Adamska I., Biernacka P. (2021). Bioactive Substances in Safflower Flowers and Their Applicability in Medicine and Health-Promoting Foods. Int. J. Food Sci..

[11] Sato S., Kusakari T., Suda T., Kasai T., Kumazawa T., Onodera J., Obara H. (2005). Efficient Synthesis of Analogs of Safflower Yellow B, Carthamin, and Its Precursor: Two Yellow and One Red Dimeric Pigments in Safflower Petals. Tetrahedron.

[12] Ji Y., Guo S., Wang B., Yu M. (2018). Extraction and Determination of Flavonoids in *Carthamus tinctorius*. Open Chem..

[13] Zhou L., Lu L., Chen C., Zhou T., Wu Q., Wen F., Chen J., Pritchard H.W., Peng C., Pei J. (2022). Comparative Changes in Sugars and Lipids Show Evidence of a Critical Node for Regeneration in Safflower Seeds during Aging. Front. Plant Sci..

[14] Wood C.C., Okada S., Taylor M.C., Menon A., Mathew A., Cullerne D., Stephen S.J., Allen R.S., Zhou X., Liu Q. (2018). Seed-specific RNAi in Safflower Generates a Superhigh Oleic Oil with Extended Oxidative Stability. Plant Biotechnol. J..

[15] Sardouei-Nasab S., Nemati Z., Mohammadi-Nejad G., Haghi R., Blattner F.R. (2023). Phylogenomic Investigation of Safflower (*Carthamus tinctorius*) and Related Species Using Genotyping-by-Sequencing (GBS). Sci. Rep..

[16] Wu Z., Liu H., Zhan W., Yu Z., Qin E., Liu S., Yang T., Xiang N., Kudrna D., Chen Y. (2021). The Chromosome-Scale Reference Genome of Safflower (*Carthamus tinctorius*) Provides Insights into Linoleic Acid and Flavonoid Biosynthesis. Plant Biotechnol. J..

[17] Li H., Dong Y., Yang J., Liu X., Wang Y., Yao N., Guan L., Wang N., Wu J., Li X. (2012). De Novo Transcriptome of Safflower and the Identification of Putative Genes for Oleosin and the Biosynthesis of Flavonoids. PLoS ONE.

[18] Scaglione D., Reyes-Chin-Wo S., Acquadro A., Froenicke L., Portis E., Beitel C., Tirone M., Mauro R., Lo Monaco A., Mauromicale G. (2016). The Genome Sequence of the Outbreeding Globe Artichoke Constructed de Novo Incorporating a Phase-Aware Low-Pass Sequencing Strategy of F1 Progeny. Sci. Rep..

[19] Fan K., Qin Y., Hu X., Xu J., Ye Q., Zhang C., Ding Y., Li G., Chen Y., Liu J. (2023). Identification of Genes Associated with Fatty Acid Biosynthesis Based on 214 Safflower Core Germplasm. BMC Genom..

[20] Cao S., Zhou X.-R., Wood C.C., Green A.G., Singh S.P., Liu L., Liu Q. (2013). A Large and Functionally Diverse Family of Fad2 Genes in Safflower (*Carthamus tinctorius* L.). BMC Plant Biol..

[21] Li D., Wang Q., Xu X., Yu J., Chen Z., Wei B., Wu W. (2021). Temporal Transcriptome Profiling of Developing Seeds Reveals Candidate Genes Involved in Oil Accumulation in Safflower (*Carthamus tinctorius* L.). BMC Plant Biol..

[22] Çulha Erdal Ş., Eyidoğan F., Ekmekçi Y. (2021). Comparative Physiological and Proteomic Analysis of Cultivated and Wild Safflower Response to Drought Stress and Re-Watering. Physiol. Mol. Biol. Plants.

[23] Shaki F., Ebrahimzadeh Maboud H., Niknam V. (2020). Differential Proteomics: Effect of Growth Regulators on Salt Stress Responses in Safflower Seedlings. Pestic. Biochem. Physiol..

[24] Bárta J., Bártová V., Jarošová M., Švajner J., Smetana P., Kadlec J., Filip V., Kyselka J., Berčíková M., Zdráhal Z. (2021). Oilseed Cake Flour Composition, Functional Properties and Antioxidant Potential as Effects of Sieving and Species Differences. Foods.

[25] Pu Z., Zhang S., Tang Y., Shi X., Tao H., Yan H., Chen J., Yue S., Chen Y., Zhu Z. (2021). Study on Changes in Pigment Composition during the Blooming Period of Safflower Based on Plant Metabolomics and Semi-quantitative Analysis. J. Sep. Sci..

[26] Kim N.S., Kim J.K., Sathasivam R., Park H.W., Nguyen B.V., Kim M.C., Cuong D.M., Chung Y.S., Park S.U. (2021). Impact of Betaine Under Salinity on Accumulation of Phenolic Compounds in Safflower (*Carthamus tinctorius* L.) Sprouts. Nat. Product. Commun..

[27] Qiang T., Liu J., Dong Y., Ma Y., Zhang B., Wei X., Liu H., Xiao P. (2020). Transcriptome Sequencing and Chemical Analysis Reveal the Formation Mechanism of White Florets in *Carthamus tinctorius* L. Plants.

[28] Ren C., Chen C., Dong S., Wang R., Xian B., Liu T., Xi Z., Pei J., Chen J. (2022). Integrated Metabolomics and Transcriptome Analysis on Flavonoid Biosynthesis in Flowers of Safflower (*Carthamus tinctorius* L.) during Colour-Transition. PeerJ.

[29] Wang R., Ren C., Dong S., Chen C., Xian B., Wu Q., Wang J., Pei J., Chen J. (2021). Integrated Metabolomics and Transcriptome Analysis of Flavonoid Biosynthesis in Safflower (*Carthamus tinctorius* L.) with Different Colors. Front. Plant Sci..

[30] Chen J., Wang J., Wang R., Xian B., Ren C., Liu Q., Wu Q., Pei J. (2020). Integrated Metabolomics and Transcriptome Analysis on Flavonoid Biosynthesis in Safflower (*Carthamus tinctorius* L.) under MeJA Treatment. BMC Plant Biol..

[31] Chen C., Wang R., Dong S., Wang J., Ren C., Chen C., Yan J., Zhou T., Wu Q., Pei J. (2022). Integrated Proteome and Lipidome Analysis of Naturally Aged Safflower Seeds Varying in Vitality. Plant Biol. J..

[32] Vincent D., Bui A., Ram D., Ezernieks V., Bedon F., Panozzo J., Maharjan P., Rochfort S., Daetwyler H., Hayden M. (2022). Mining the Wheat Grain Proteome. Int. J. Mol. Sci..

[33] Vincent D., Bui A., Ezernieks V., Shahinfar S., Luke T., Ram D., Rigas N., Panozzo J., Rochfort S., Daetwyler H. (2022). A Community Resource to Mass Explore the Wheat Grain Proteome and Its Application to the Late-Maturity Alpha-Amylase (LMA) Problem. GigaScience.

[34] Holman J.D., Tabb D.L., Mallick P. (2014). Employing ProteoWizard to Convert Raw Mass Spectrometry Data. Curr. Protoc. Bioinform..

[35] Creasy D.M., Cottrell J.S. (2002). Error Tolerant Searching of Uninterpreted Tandem Mass Spectrometry Data. Proteomics.

[36] Wishart D.S., Guo A., Oler E., Wang F., Anjum A., Peters H., Dizon R., Sayeeda Z., Tian S., Lee B.L. (2022). HMDB 5.0: The Human Metabolome Database for 2022. Nucleic Acids Res..

[37] Reddy P., Plozza T., Ezernieks V., Stefanelli D., Scalisi A., Goodwin I., Rochfort S. (2022). Metabolic Pathways for Observed Impacts of Crop Load on Floral Induction in Apple. Int. J. Mol. Sci..

[38] Pang Z., Chong J., Zhou G., de Lima Morais D.A., Chang L., Barrette M., Gauthier C., Jacques P.-É., Li S., Xia J. (2021). MetaboAnalyst 5.0: Narrowing the Gap between Raw Spectra and Functional Insights. Nucleic Acids Res..

[39] Djoumbou Feunang Y., Eisner R., Knox C., Chepelev L., Hastings J., Owen G., Fahy E., Steinbeck C., Subramanian S., Bolton E. (2016). ClassyFire: Automated Chemical Classification with a Comprehensive, Computable Taxonomy. J. Cheminform..

[40] The UniProt Consortium (2018). UniProt: The Universal Protein Knowledgebase. Nucleic Acids Res..

[41] Cock P.J.A., Chilton J.M., Grüning B., Johnson J.E., Soranzo N. (2015). NCBI BLAST+ Integrated into Galaxy. GigaScience.

[42] Supek F., Bošnjak M., Škunca N., Šmuc T. (2011). REVIGO Summarizes and Visualizes Long Lists of Gene Ontology Terms. PLoS ONE.

[43] Kanehisa M., Sato Y. (2020). KEGG Mapper for Inferring Cellular Functions from Protein Sequences. Protein Sci..

[44] Kanehisa M., Sato Y., Kawashima M. (2022). KEGG Mapping Tools for Uncovering Hidden Features in Biological Data. Protein Sci..

[45] Liu T., Salguero P., Petek M., Martinez-Mira C., Balzano-Nogueira L., Ramšak Ž., McIntyre L., Gruden K., Tarazona S., Conesa A. (2022). PaintOmics 4: New Tools for the Integrative Analysis of Multi-Omics Datasets Supported by Multiple Pathway Databases. Nucleic Acids Res..

[46] (2023). Plant Metabolics Network (PMN). https://pmn.plantcyc.org/Organism-Summary?object=ARA.

[47] Ahmadi S., Winter D., Wang X., Kuruc M. (2019). Identification of Unexpected Protein Modifications by Mass Spectrometry-Based Proteomics. Functional Proteomics.

[48] Bockisch M. (1998). Vegetable Fats and Oils. Fats and Oils Handbook.

[49] Zhang Q., Hu N., Li W., Ding C., Ma T., Bai B., Wang H., Suo Y., Wang X., Ding C. (2015). Preparative Separation of N-Feruloyl Serotonin and N-(p-Coumaroyl) Serotonin from Safflower Seed Meal Using High-Speed Counter-Current Chromatography. J. Chromatogr. Sci..

[50] Nazeam J.A., El-Hefnawy H.M., Omran G., Singab A.-N. (2018). Chemical Profile and Antihyperlipidemic Effect of *Portulaca oleracea* L. Seeds in Streptozotocin-Induced Diabetic Rats. Nat. Prod. Res..

[51] Li Q., Wang B.-C., Xu Y., Zhu Y.-X. (2007). Systematic Studies of 12S Seed Storage Protein Accumulation and Degradation Patterns during *Arabidopsis* Seed Maturation and Early Seedling Germination Stages. J. Biochem. Mol. Biol..

[52] D’Agostino N., Buonanno M., Ayoub J., Barone A., Monti S.M., Rigano M.M. (2019). Identification of Non-Specific Lipid Transfer Protein Gene Family Members in *Solanum Lycopersicum* and Insights into the Features of Sola l 3 Protein. Sci. Rep..

[53] Liu F., Zhang X., Lu C., Zeng X., Li Y., Fu D., Wu G. (2015). Non-Specific Lipid Transfer Proteins in Plants: Presenting New Advances and an Integrated Functional Analysis. J. Exp. Bot..

[54] Lees J.A., Reinisch K.M. (2020). Inter-Organelle Lipid Transfer: A Channel Model for Vps13 and Chorein-N Motif Proteins. Curr. Opin. Cell Biol..

[55] Boatright J., Negre F., Chen X., Kish C.M., Wood B., Peel G., Orlova I., Gang D., Rhodes D., Dudareva N. (2004). Understanding in Vivo Benzenoid Metabolism in *Petunia* Petal Tissue. Plant Physiol..

[56] Mostafa S., Wang Y., Zeng W., Jin B. (2022). Floral Scents and Fruit Aromas: Functions, Compositions, Biosynthesis, and Regulation. Front. Plant Sci..

[57] Paniagua C., Bilkova A., Jackson P., Dabravolski S., Riber W., Didi V., Houser J., Gigli-Bisceglia N., Wimmerova M., Budínská E. (2017). Dirigent Proteins in Plants: Modulating Cell Wall Metabolism during Abiotic and Biotic Stress Exposure. J. Exp. Bot..

[58] Hsieh K., Huang A.H.C. (2005). Lipid-Rich Tapetosomes in Brassica Tapetum Are Composed of Oleosin-Coated Oil Droplets and Vesicles, Both Assembled in and Then Detached from the Endoplasmic Reticulum. Plant J..

[59] Bannenberg G., Martínez M., Hamberg M., Castresana C. (2009). Diversity of the Enzymatic Activity in the Lipoxygenase Gene Family of *Arabidopsis Thaliana*. Lipids.

[60] Mueller L.A., Zhang P., Rhee S.Y. (2003). AraCyc: A Biochemical Pathway Database for *Arabidopsis*. Plant Physiol..

[61] Floerl S., Majcherczyk A., Possienke M., Feussner K., Tappe H., Gatz C., Feussner I., Kües U., Polle A. (2012). *Verticillium Longisporum* Infection Affects the Leaf Apoplastic Proteome, Metabolome, and Cell Wall Properties in *Arabidopsis Thaliana*. PLoS ONE.

[62] López-Hidalgo C., Guerrero-Sánchez V.M., Gómez-Gálvez I., Sánchez-Lucas R., Castillejo-Sánchez M.A., Maldonado-Alconada A.M., Valledor L., Jorrín-Novo J.V. (2018). A Multi-Omics Analysis Pipeline for the Metabolic Pathway Reconstruction in the Orphan Species *Quercus Ilex*. Front. Plant Sci..

[63] García-Alcalde F., García-López F., Dopazo J., Conesa A. (2011). Paintomics: A Web Based Tool for the Joint Visualization of Transcriptomics and Metabolomics Data. Bioinformatics.

[64] Chong J., Wishart D.S., Xia J. (2019). Using MetaboAnalyst 4.0 for Comprehensive and Integrative Metabolomics Data Analysis. Curr. Protoc. Bioinform..

[65] Wang W.-Q., Liu S.-J., Song S.-Q., Møller I.M. (2015). Proteomics of Seed Development, Desiccation Tolerance, Germination and Vigor. Plant Physiol. Biochem..

[66] Domergue J.-B., Abadie C., Limami A., Way D., Tcherkez G. (2019). Seed Quality and Carbon Primary Metabolism. Plant Cell Environ..

