# Supplementary material for: Integrated Proteomics and Metabolomics of Safflower Petal Wilting and Seed Development
Source: Biomolecules. 2024 Mar 28;14(4):414. doi: 10.3390/biom14040414 (PMC11048707; doi:10.3390/biom14040414)
Supplement: Supplementary file 1 [file biomolecules-14-00414-s001.zip › Vincent-et-al_Suppl-Figures_2024-03-14.pptx]

## Slide 1
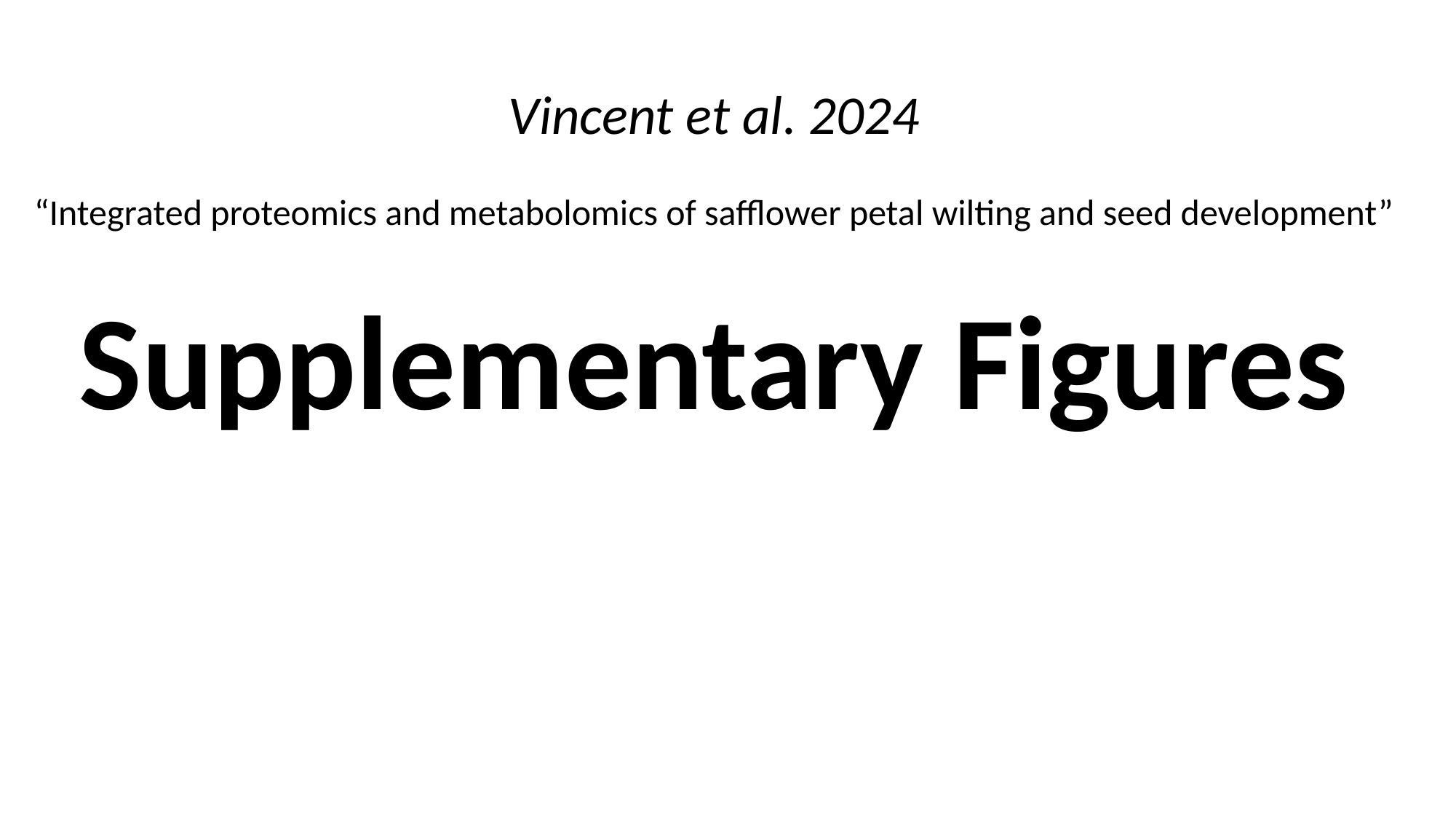

Vincent et al. 2024
“Integrated proteomics and metabolomics of safflower petal wilting and seed development”
Supplementary Figures

## Slide 2
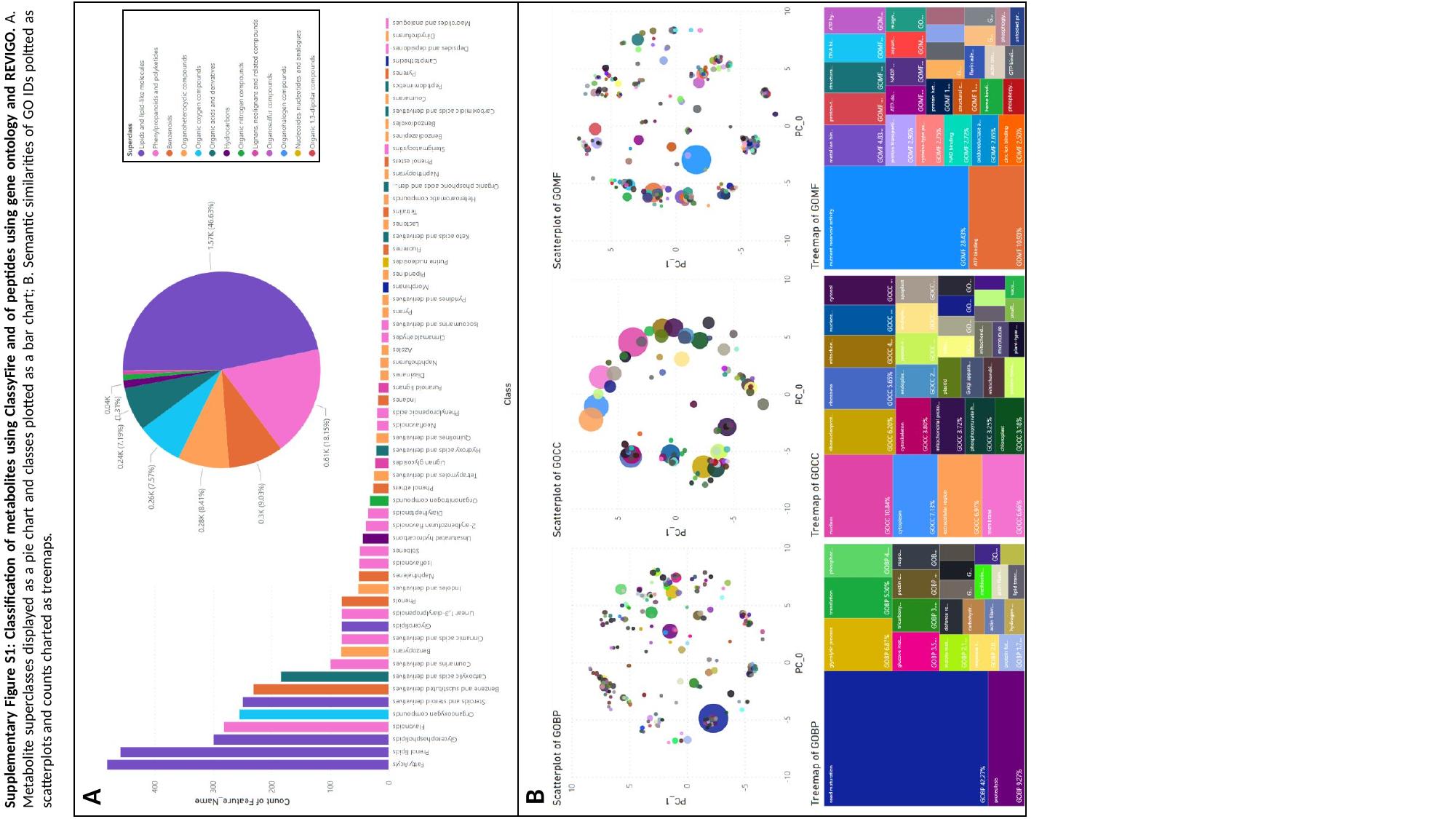

A
B
Supplementary Figure S1: Classification of metabolites using ClassyFire and of peptides using gene ontology and REVIGO. A. Metabolite superclasses displayed as a pie chart and classes plotted as a bar chart; B. Semantic similarities of GO IDs poltted as scatterplots and counts charted as treemaps.

## Slide 3
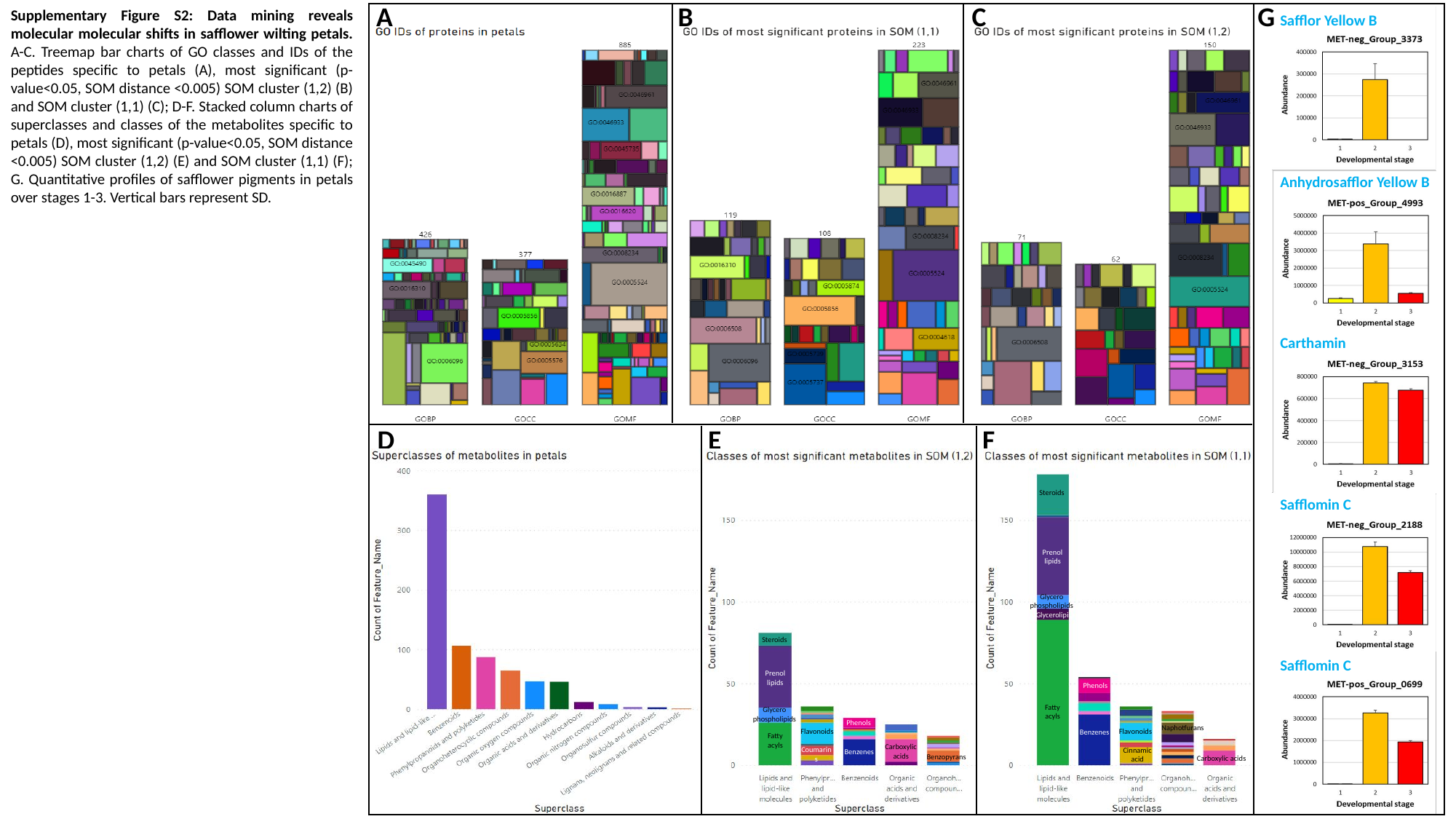

Supplementary Figure S2: Data mining reveals molecular molecular shifts in safflower wilting petals. A-C. Treemap bar charts of GO classes and IDs of the peptides specific to petals (A), most significant (p-value<0.05, SOM distance <0.005) SOM cluster (1,2) (B) and SOM cluster (1,1) (C); D-F. Stacked column charts of superclasses and classes of the metabolites specific to petals (D), most significant (p-value<0.05, SOM distance <0.005) SOM cluster (1,2) (E) and SOM cluster (1,1) (F); G. Quantitative profiles of safflower pigments in petals over stages 1-3. Vertical bars represent SD.
A
B
C
G
Safflor Yellow B
Anhydrosafflor Yellow B
Carthamin
Safflomin C
Safflomin C
D
E
F
Steroids
Prenol lipids
Glycero
phospholipids
Glycerolipids
Steroids
Prenol lipids
Phenols
Fatty acyls
Glycero
phospholipids
Phenols
Naphotfurans
Flavonoids
Flavonoids
Benzenes
Fatty acyls
Carboxylic acids
Coumarins
Cinnamic acid
Benzenes
Benzopyrans
Carboxylic acids

## Slide 4
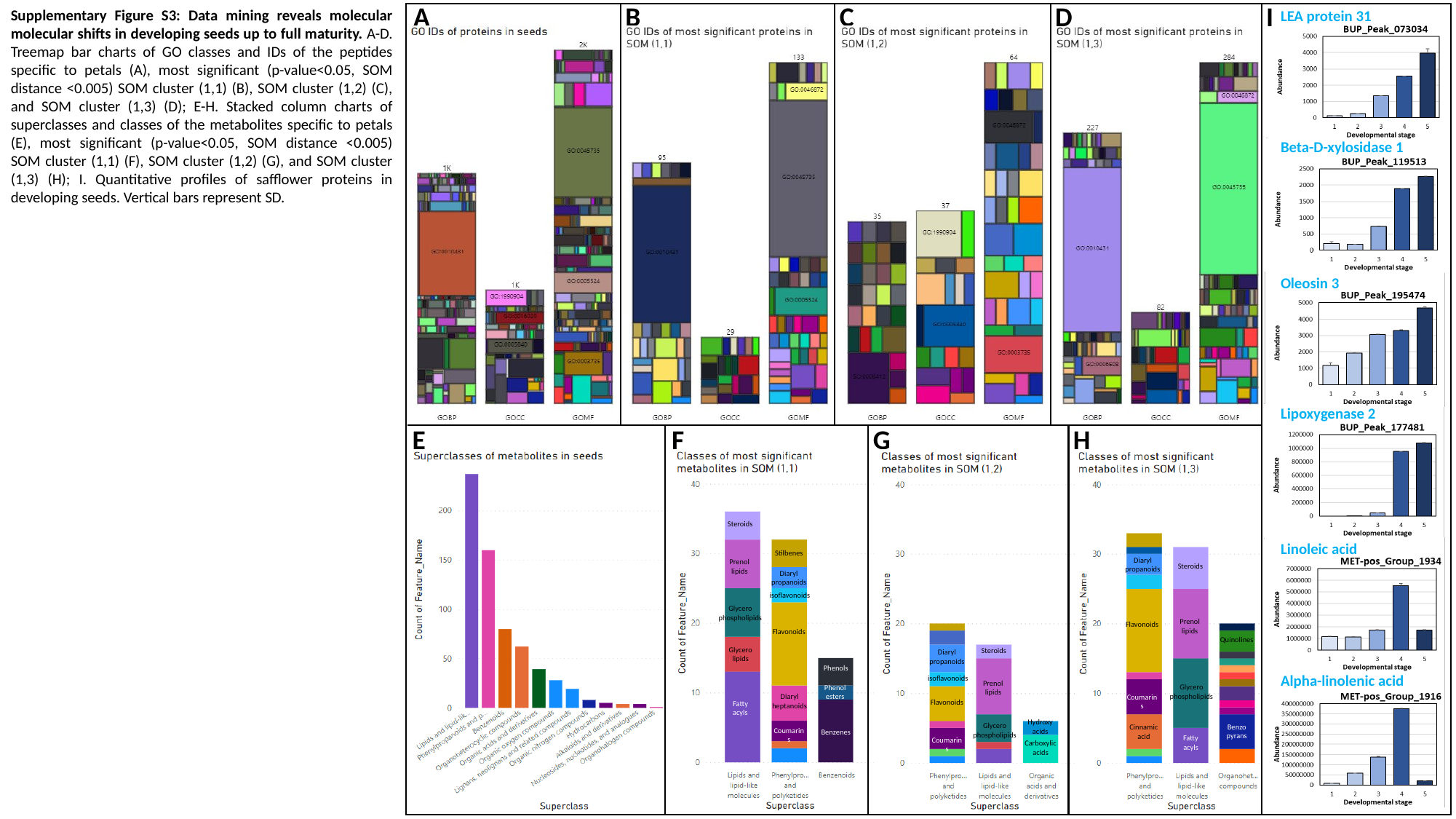

Supplementary Figure S3: Data mining reveals molecular molecular shifts in developing seeds up to full maturity. A-D. Treemap bar charts of GO classes and IDs of the peptides specific to petals (A), most significant (p-value<0.05, SOM distance <0.005) SOM cluster (1,1) (B), SOM cluster (1,2) (C), and SOM cluster (1,3) (D); E-H. Stacked column charts of superclasses and classes of the metabolites specific to petals (E), most significant (p-value<0.05, SOM distance <0.005) SOM cluster (1,1) (F), SOM cluster (1,2) (G), and SOM cluster (1,3) (H); I. Quantitative profiles of safflower proteins in developing seeds. Vertical bars represent SD.
A
B
C
D
I
LEA protein 31
Beta-D-xylosidase 1
Oleosin 3
Lipoxygenase 2
E
F
G
H
Linoleic acid
Alpha-linolenic acid
Steroids
Stilbenes
Diaryl
propanoids
Prenol lipids
Steroids
Diaryl
propanoids
isoflavonoids
Glycero
phospholipids
Prenol lipids
Flavonoids
Flavonoids
Quinolines
Glycero
lipids
Steroids
Diaryl
propanoids
Phenols
isoflavonoids
Prenol lipids
Glycero
phospholipids
Phenol esters
Diaryl
heptanoids
Coumarins
Flavonoids
Fatty acyls
Hydroxy acids
Glycero
phospholipids
Benzo
pyrans
Cinnamic acid
Coumarins
Benzenes
Fatty acyls
Coumarins
Carboxylic acids

## Slide 5
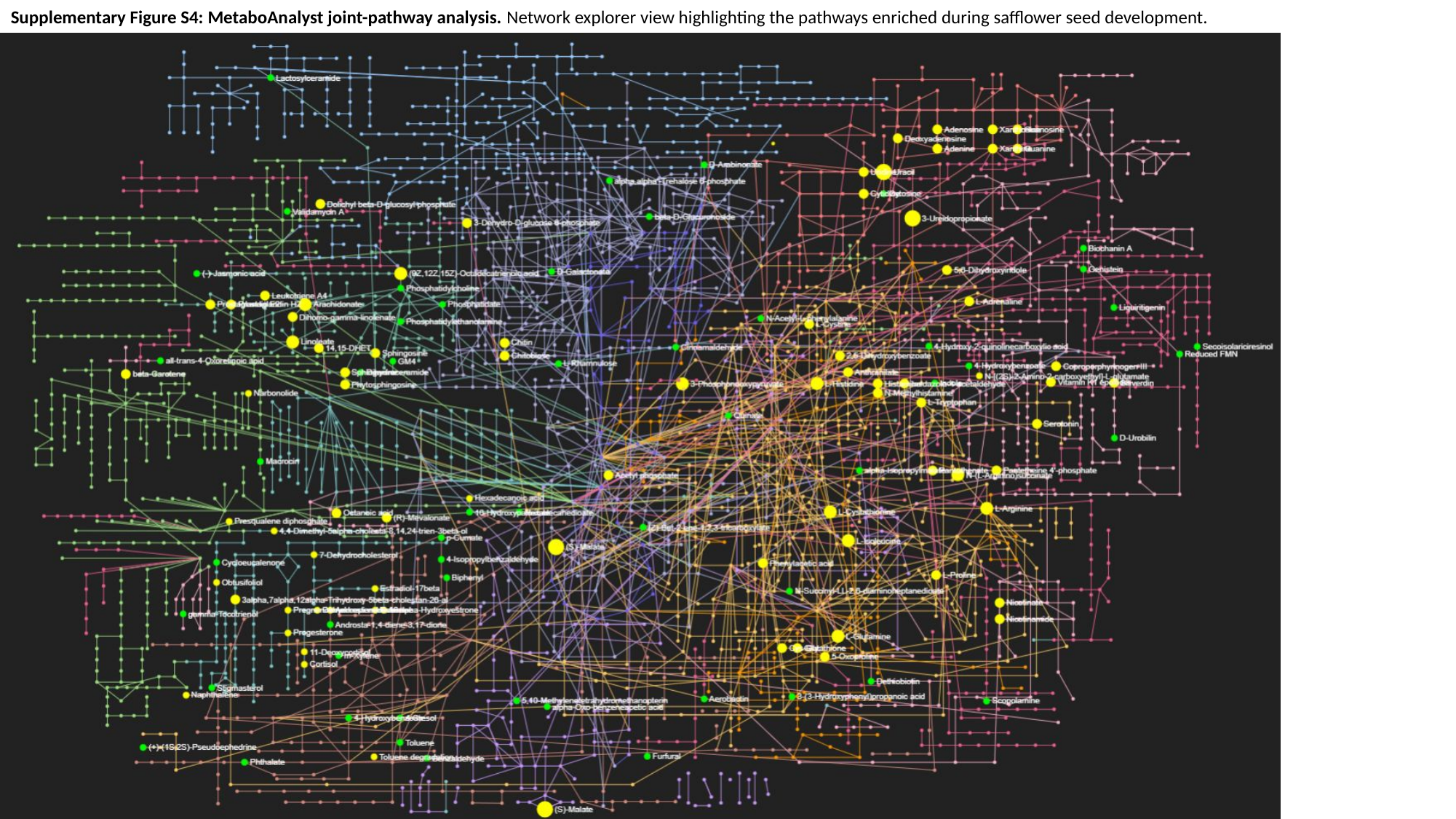

Supplementary Figure S4: MetaboAnalyst joint-pathway analysis. Network explorer view highlighting the pathways enriched during safflower seed development.

## Slide 6
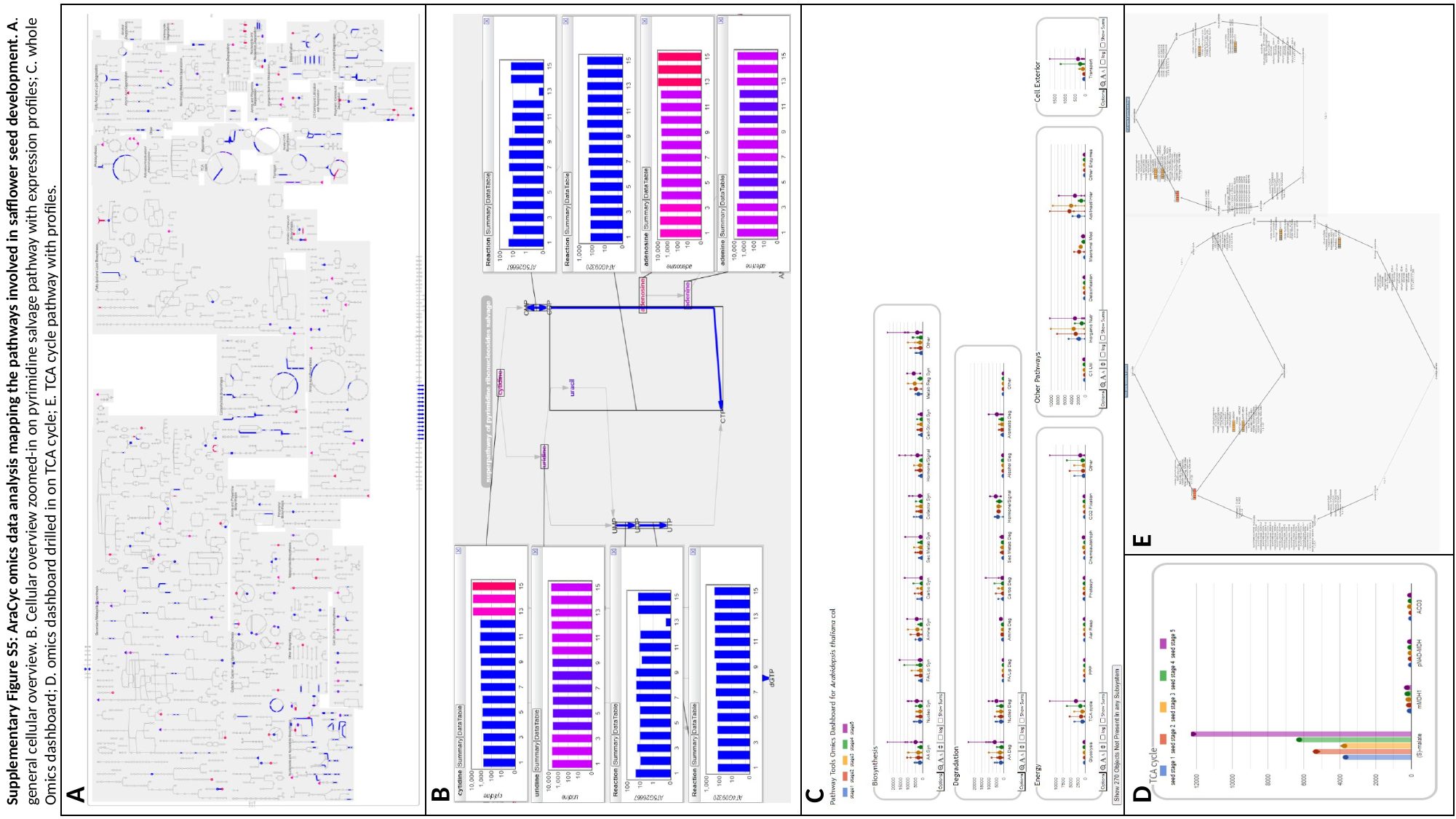

E
D
A
B
C
Supplementary Figure S5: AraCyc omics data analysis mapping the pathways involved in safflower seed development. A. general cellular overview. B. Cellular overview zoomed-in on pyrimidine salvage pathway with expression profiles; C. whole Omics dashboard; D. omics dashboard drilled in on TCA cycle; E. TCA cycle pathway with profiles.

## Slide 7
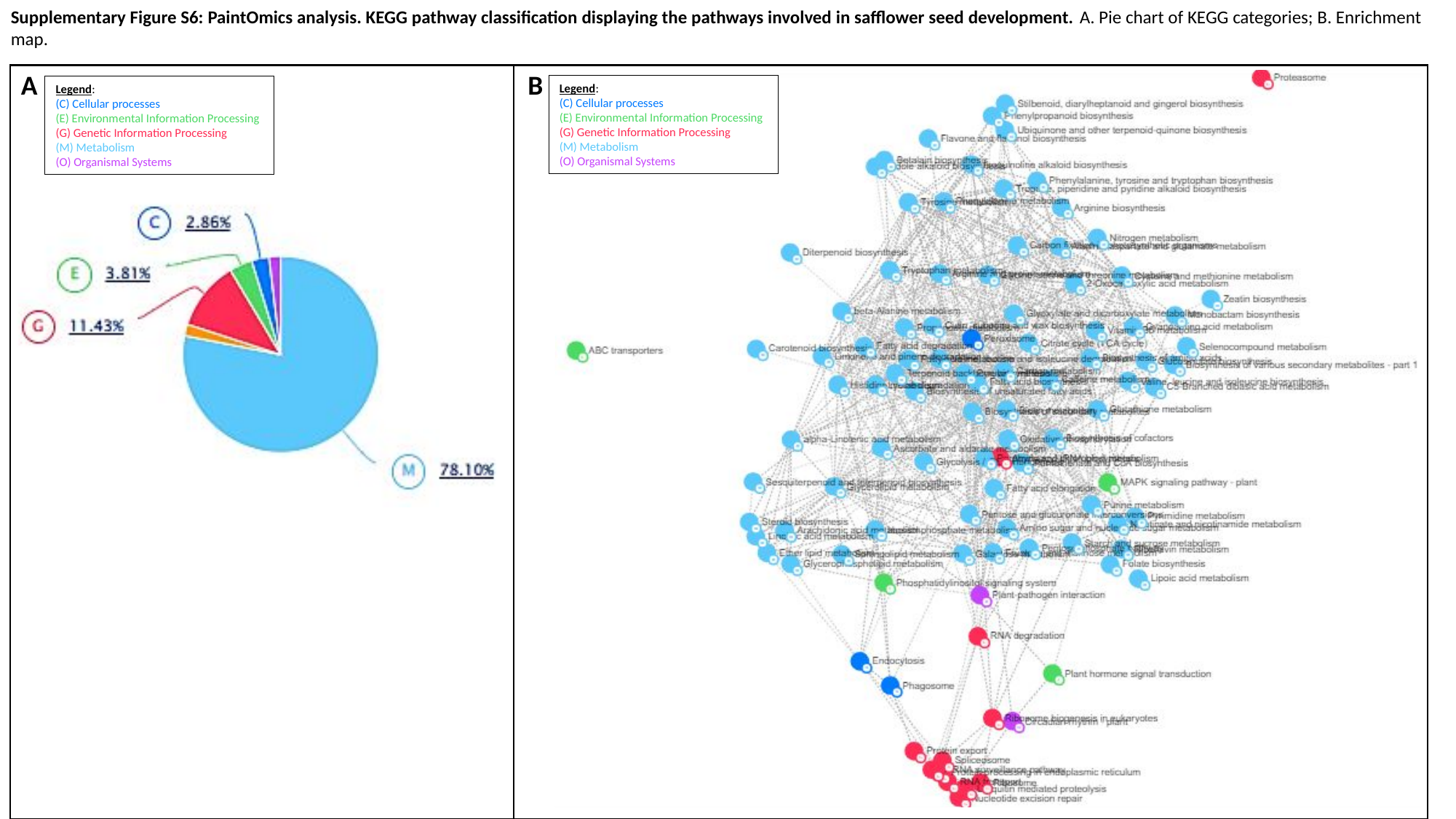

Supplementary Figure S6: PaintOmics analysis. KEGG pathway classification displaying the pathways involved in safflower seed development. A. Pie chart of KEGG categories; B. Enrichment map.
A
B
Legend:
(C) Cellular processes
(E) Environmental Information Processing
(G) Genetic Information Processing
(M) Metabolism
(O) Organismal Systems
Legend:
(C) Cellular processes
(E) Environmental Information Processing
(G) Genetic Information Processing
(M) Metabolism
(O) Organismal Systems
